# Prioritizing Artificial Intelligence Opportunities for Food as Health in the U.S. Agrifood Value Chain

**DOI:** 10.3390/foods15183215

**Published:** 2026-09-11

**Authors:** Lourival Carmo Monaco Neto, Allan W. Gray

**Affiliations:** Department of Agricultural Economics, College of Agriculture, Purdue University, West Lafayette, IN 47907, USA; gray@purdue.edu

**Keywords:** artificial intelligence, food as health, opportunity mapping, jobs-to-be-done, systematic review

## Abstract

Food and health are converging across the agrifood value chain, and artificial intelligence (AI) is widely promoted to operationalize the link, yet firms cannot tell which food-as-health opportunities current AI can address. We identify the agrifood industry’s highest-priority food-as-health opportunities in the United States and assess, against the peer-reviewed AI literature, how far current AI can address each. A PRISMA 2020 systematic review of 34 U.S.-focused studies establishes the problem space; a Jobs-to-Be-Done map organizes ninety opportunity areas; a blind discovery forum with 37 senior U.S. practitioners gives an independent reading; a convergence analysis joins the map and the forum; and a separate assessment grades each opportunity against the AI literature. Practitioner priorities converge on twelve opportunities in the two segments that physically determine food’s health attributes, agricultural production and food manufacturing. These resolve into five AI capability families. The evidence is strong for sensing, traceability, design, and personalization, and more qualified for evidence synthesis and regulatory intelligence, where reliability limits require human oversight. One core need lies beyond AI, a preliminary finding of a symmetric long-term-contract gap that appears relational. We grade an agenda by how far current AI reaches, with an explicit boundary on what it cannot address.

## 1. Introduction

The convergence of food and health has shifted from a public health aspiration to a commercial reality that is reshaping competitive position across the agrifood value chain. Diet-related noncommunicable diseases are among the leading contributors to global mortality [1], food-based interventions have measurable effects on cardiovascular disease, diabetes, and obesity [2], and many United States consumers actively try to improve their diets [3]. The institutional scaffolding has followed: the Food is Medicine framework has been formalized in clinical practice [4], produce prescription programs have moved from local pilots to coordinated national initiatives [5,6], and the diffusion of GLP-1 receptor agonist medications is altering household food purchases in ways that manufacturers and retailers are only beginning to absorb [7,8]. Firms across the chain, from input suppliers to branded manufacturers, are repositioning portfolios in response.

At the same time, artificial intelligence has become the technology most often invoked to operationalize the food–health link. The applied literature clusters into five recurring capability families, namely sensing and measurement, traceability and verification, evidence synthesis and regulatory intelligence, generative and predictive design, and personalized nutrition, documented as accelerants across food discovery and product innovation [9], precision and personalized nutrition [10,11], food processing and manufacturing [12], and supply-chain traceability and safety [13,14]. The promise is real but diffuse: AI is presented as relevant to nearly everything, which leaves firms without a basis for deciding which of these capabilities to apply to the opportunities that actually matter. The question we take up is which of the five current AI can credibly deliver, and where.

These movements pose a strategic question that the existing literature does not answer. Agrifood firms must decide where, among many plausible responses, food-as-health investment creates durable advantage, and then which of those opportunities current AI can credibly address. Biofortified seeds, regenerative production, nutrient-preserving processing, medically supportive products, and transparency platforms each address a piece of the problem, but each draws on different capabilities and faces different competitive dynamics [15]. The scholarship treats food and health largely as a public health, nutrition science, or sustainability problem [16,17], and the rapidly growing AI-in-food literature is organized by technology rather than by where value is created in the chain. What is missing is a view of the opportunity space across the full value chain, read against an independent practitioner reading, and connected explicitly to the AI capabilities that could act on it.

This study takes up that gap. Our goal is to identify where the most strongly supported food-as-health opportunities lie for agrifood firms, what makes them so, and how far current AI can help firms act on them. Four questions organize the study. First, what does the peer-reviewed literature establish about how food-as-health value is created and constrained across the chain? Second, which opportunities do senior practitioners surface as most pressing when they describe their own jobs, pains, and gains? Third, where do the literature-grounded and practitioner-grounded views converge, and what structural patterns govern where opportunity concentrates? Fourth, which of the prioritized opportunities can current AI address, and where does its reach end?

We used four complementary methods, each supplying what the others cannot. A systematic review maps the problem space and grounds the analysis in transparent evidence. A Jobs-to-Be-Done opportunity map turns that evidence into a structured field of candidate opportunities tied to value chain positions. A blind practitioner discovery forum gives an independent, industry-defined reading of the same space. We then bring the literature-grounded map and the practitioner-grounded landscape together to identify the most strongly supported opportunities, and we assess each against the peer-reviewed AI literature to establish which ones current AI can address. Innovation analysis specifies what needs to be done; the AI literature specifies how, but only where the evidence supports it.

We make three contributions. First, we map each priority opportunity to the AI capability family that can address it and grade the strength of the peer-reviewed evidence, separating what current AI can deliver now, what it can support only under human oversight, and where the binding constraint lies outside computation. Second, we deliver the priority set itself: twelve food-as-health opportunities supported by both literature and practice and organized around four cross-segment platform needs. Third, we show that food-as-health innovation is value-origination-led, concentrated in the two segments that physically determine the health attributes of food and propagating to the remaining segments as derived demand [15,18]. The paper proceeds as follows. Section 2 develops the conceptual background and the five AI capability families. Section 3 describes the research design. Section 4 reports where opportunity concentrates. Section 5, the analytical payoff, assesses how far current AI can address the priority opportunities. Section 6 discusses implications, limitations, and future research, Section 7 sets out future perspectives, and Section 8 concludes.

## 2. Conceptual Background

Our analysis rests on four bodies of work: food as a domain of agribusiness strategy, Jobs-to-Be-Done theory as a lens for locating opportunity, value chain analysis as the structure that ties opportunity to organizational position, and the AI capabilities through which firms can act on the resulting opportunities. We introduce each for the role it plays in the inquiry rather than as a standalone review.

### 2.1. Food as a Domain of Agribusiness Strategy

Food and health are linked at every stage of the agrifood value chain, but the strategic question of where firms can most effectively act on that linkage has received little attention. Production decisions shape nutritional composition before any downstream transformation [19,20]; processing either preserves or degrades that content [21,22]; manufacturing determines what nutritional value ultimately reaches the consumer [4]; distribution and access govern whether nutritious food reaches the populations whose health is at stake [23,24]; and consumer perception translates available food into actual diet [25,26]. Public health and nutrition science characterize each stage individually, but the integrated question of where in the chain value is created, and where it can be captured, remains open. Three forces have raised the stakes. Nutrition-sensitive agriculture has matured from a development-policy framing [27] into a planetary-health agenda with documented impact pathways [16,17]. The infrastructure for treating food as a healthcare intervention has formalized [4,6,28]. And consumer demand is shifting under regulatory, cultural, and pharmacological pressure [7,8]. The combined effect is a domain in which the commercial opportunity is real but unstructured.

### 2.2. Jobs-to-Be-Done and the Meta-Job Construct

Jobs-to-Be-Done theory reframes innovation around the persistent progress that actors seek, treating the job rather than the product as the unit of analysis [29], and Outcome-Driven Innovation operationalizes it by ranking jobs on importance and current satisfaction so that underserved outcomes surface as the strongest opportunities [30]. Standard applications assume one firm serving one customer, which does not hold in a value chain where a single consumer-defined need is met through the coordinated action of many actors. The meta-job construct resolves this by defining a stable functional challenge that recurs across actors but manifests differently by position [31], organizing the opportunity landscape across the chain while preserving the segment-level differentiation that strategy requires. We operationalize jobs at the functional level; the social and emotional jobs present in the forum data we do not treat as separate opportunity coordinates.

### 2.3. Value Chain Structure: Primary and Supporting Segments

Value chain analysis traces how value is created and captured as products move from inputs to consumers [18,32], and the agribusiness literature identifies six agrifood segments: input manufacturing, input distribution, agricultural production, processing and handling, food manufacturing, and support services [15,33]. Two strategy distinctions guide where food-as-health opportunity should concentrate: Porter [32] separates primary activities, which create and transform the product, from supporting activities that enable them, and Outcome-Driven Innovation parallels this with job executors and job supporters [30]. Both imply that the segments most directly determining the health attributes of food, agricultural production and food manufacturing, are primary, while the other four are supporting and derive their food-as-health activity from primary-segment requirements. We treat this designation as a working proposition and examine it against the evidence; Section 4.2 reports how far the systematic review bears it out.

The distinction between primary and supporting segments describes where value originates. A second distinction describes the character of the opportunities themselves. Within any segment, an opportunity may be proactive, originating health value through the firm’s own decisions, or reactive, responding to and enabling demand that originates elsewhere in the chain. The two distinctions are related but not identical, and keeping them separate avoids a common imprecision: it is not that opportunities are absent from the supporting segments, but that the opportunities located there are predominantly reactive, taking their shape from the requirements of the primary segments. This proactive and reactive framing is used throughout the analysis to characterize individual opportunities. The reactive characterization reflects the demand-focused lens adopted here and does not deny that supporting-segment actors can also originate value through technology push, as when input firms introduce new seed traits that create their own demand [34].

### 2.4. Artificial Intelligence as the Enabling Layer for Food-as-Health Opportunity

If innovation analysis identifies where firms should act, artificial intelligence is increasingly the means through which they act, and the capabilities relevant to food as health fall into five families that recur across the literature. The first is sensing, measurement, and prediction, in which machine learning applied to spectroscopic, hyperspectral, and remote-sensing data estimates nutritional and quality attributes non-destructively and at scale [35,36,37], and in which genomic selection and model-assisted breeding accelerate the development of nutritionally improved varieties [38,39]. The second is traceability and verification, in which AI combined with the Internet of Things and distributed-ledger technologies supports provenance tracking, authentication, fraud detection, and predictive food-safety risk assessment [13,14,40]. The third is evidence synthesis and regulatory intelligence, in which natural language processing and large language models accelerate biomedical evidence review and the parsing of regulatory text, though with documented reliability limits [41,42,43]. The fourth is generative and predictive design, in which machine learning supports reformulation, ingredient substitution, and the development of novel and alternative-protein products [9,12,44,45]. The fifth is personalized nutrition and decision support, in which AI translates individual biological and behavioral data into dietary recommendations and clinical decision support [10,11,46]. These families are an organizing taxonomy drawn from the cited AI reviews rather than an inductively derived result, and they define the solution space against which the prioritized opportunities are assessed in Section 5.

### 2.5. The Gap and What the Background Licenses

Public health and nutrition science establish the diet-health linkage and the case for food-based intervention [2,47], strategic-management research characterizes innovation in agrifood firms [15], and a large applied literature documents AI capabilities in food and agriculture [9], but no existing work integrates a transparent literature foundation, a value-chain-segmented map of opportunity, an independent practitioner reading, and a structured assessment of AI applicability into a single account of where firms should act and how AI can help them. Because the opportunity space is poorly structured, it calls for a systematic rather than purposive evidence base. Because opportunity must be tied to organizational position, it calls for the meta-job construct and the six-segment chain. Because the literature can identify candidate opportunities but not their commercial urgency, it calls for an independent practitioner layer and a way to bring the two together. And because the value of an opportunity depends partly on whether it can be acted upon, it calls for an explicit, evidence-based assessment of the AI capabilities available to address it. The design we describe next follows from these requirements.

## 3. Materials and Methods

**Figure 1:**
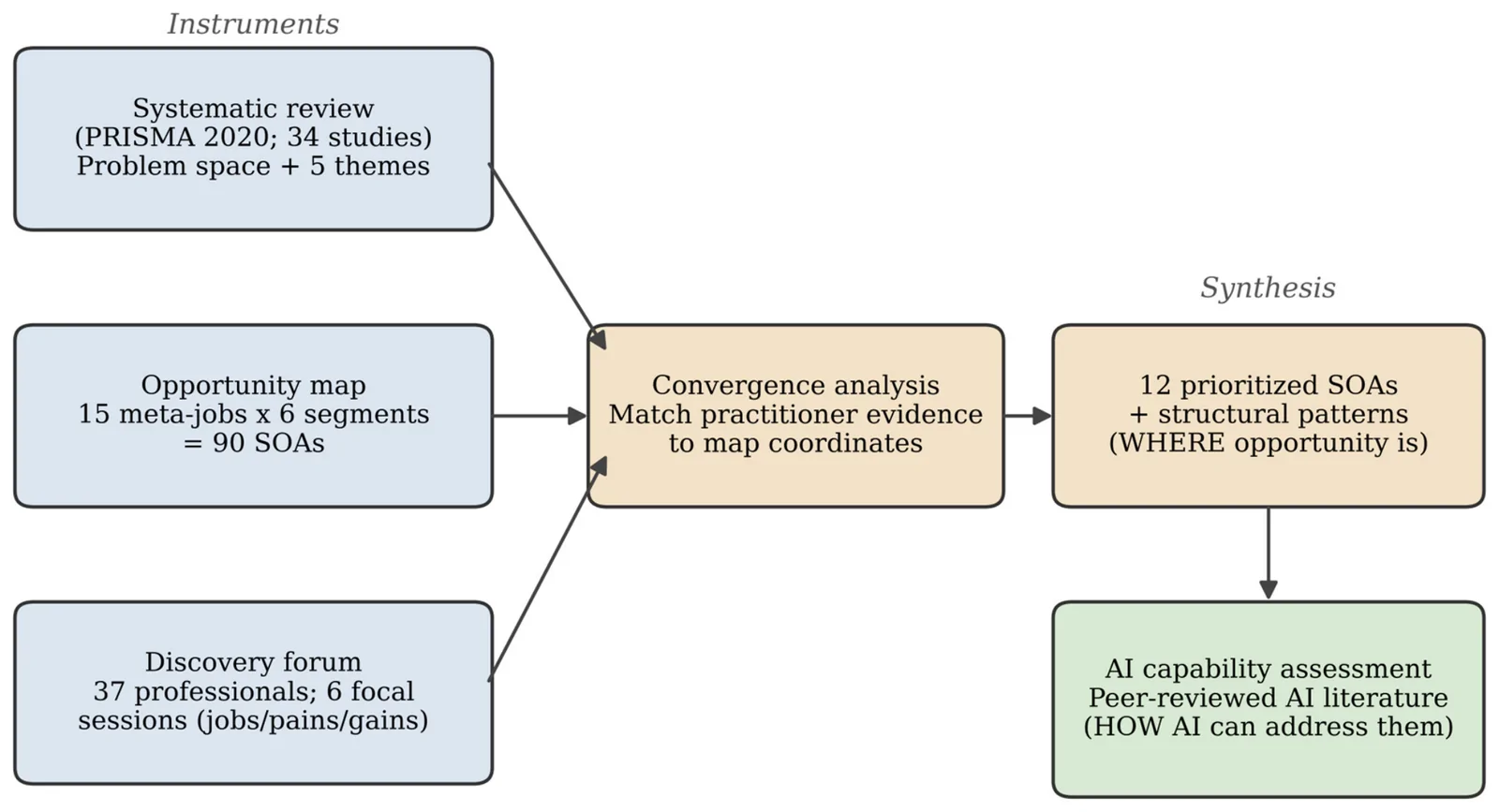
The research design. A systematic review establishes the problem space and grounds an opportunity map; a blind practitioner discovery forum supplies an independent reading of the same space; a convergence analysis matches the two to identify the prioritized opportunity set; and a structured AI capability assessment evaluates how current AI can address that set.

We used four complementary methods directed at a single goal, choosing each to supply what the others cannot. The systematic review (Section 3.1) establishes the problem space and the evidentiary base. The opportunity map (Section 3.2) translates that base into a structured field of candidate opportunities. The discovery forum (Section 3.3) gives an independent, practitioner-defined reading of the same space. The convergence analysis (Section 3.4) brings the literature-grounded and practitioner-grounded views together, and the AI capability assessment (Section 3.5) evaluates the prioritized opportunities against the peer-reviewed AI literature. Our two principal sources of evidence for where opportunity concentrates are the map and the forum; the review is the conceptual foundation on which we build the map. Figure 1 summarizes the design from inputs to outputs.

### 3.1. Establishing the Problem Space: Systematic Review

We conducted a systematic review according to the PRISMA 2020 reporting guidelines [48] to establish what the peer-reviewed literature reveals about how food-as-health value is created and constrained across the agrifood chain. The review addressed a single question: what does the peer-reviewed literature establish about how food-as-health value is created and constrained across the agrifood value chain? We searched seven databases for English-language work published between January 2015 and May 2025: PubMed, Scopus, Web of Science, Google Scholar, AGRIS, CAB Direct, and Embase. We included Google Scholar as a supplementary source, consistent with PRISMA 2020 guidance on supplementary identification methods, to capture high-impact and cross-disciplinary work not consistently indexed elsewhere, and we used targeted citation searching within Google Scholar to ensure that foundational nutrition-sensitive agriculture studies were represented. We included a study if it concerned food as health and either took the perspective of the agrifood value chain or examined how the chain influences, or is influenced by, food-as-health outcomes, was an empirical study, systematic review, or established institutional report, and was published in English within the period. To align the evidence base with the United States practitioner population we engaged later in the study, we restricted the corpus to work focused on the United States or a demonstrably United States-applicable context. The seven database searches returned 227 records, and targeted citation searching within Google Scholar added two foundational nutrition-sensitive agriculture studies, for 229 records identified in total. A team of five reviewers screened them under a distributed protocol: each reviewer was assigned a subset of the databases and screened its records against the inclusion criteria, recording include, exclude, or uncertain decisions in a shared instrument, and the team resolved inclusion by consensus, bringing uncertain records and full-text disagreements to joint discussion. To quantify the reliability of this screening, we later carried out a post hoc check: two members of the research team independently re-screened a random sample of 50 of the identified records against the five inclusion criteria, blind to one another and to the original decisions. Treating the screening decision as the binary choice on which the review turned, advance to full text (an include or uncertain record) versus exclude, agreement was moderate (Cohen’s κ = 0.56; 90.0% observed agreement; 95% CI 0.22–0.90), indicating that the criteria were applied consistently. Thirty-four studies were retained. For each retained study we extracted the first author, year, journal, study design, the specific food-as-health focus, the relevant value chain position, and the coded theme or themes. The same five reviewers then coded the corpus inductively [49], following the synthesis-without-meta-analysis principles appropriate to a heterogeneous corpus [50] and progressing through three rounds from 96 initial codes to eight intermediate categories to five final themes, with a study able to be assigned to more than one theme. Coding decisions were reached by team consensus rather than by parallel independent double-coding, an accepted basis for interpretive synthesis where the aim is a shared reading rather than the measurement of a fixed construct [51,52]. The full search strings, the PRISMA flow diagram, the 34-study characteristics table, and the coding progression are in the Supplementary Materials. We appraised the methodological quality of the included studies with design-appropriate tools, reported in Supplementary S9.

### 3.2. Translating Evidence into Opportunities: The Map

We built the opportunity map from the five themes in three steps. First, we decomposed each theme into meta-jobs, each a stable functional challenge expressed from the actor’s perspective, stable under changes in solution, and differentiable across segments; this yielded three meta-jobs per theme and fifteen in total (Table 1), three being the point at which further candidates duplicated existing jobs or failed the criteria. Second, we crossed the fifteen meta-jobs with the six segments, each cell defining one Strategic Opportunity Area (SOA) and the full matrix defining ninety SOAs; the ninety cells are an analytical scaffold for locating opportunity, not ninety independently evidenced findings. Third, we documented each SOA with a consistent template (rationale, bounded definition, underlying jobs, examples, and resources), following the mapping technique established for climate-smart agrifood systems [31] and enriched through targeted searching of industry sources where the published literature does not describe a cell in commercial terms. The template and the full ninety-SOA documentation are in the Supplementary Materials (S4). We checked the fifteen meta-jobs and ninety SOAs for internal consistency and for coverage of the five themes, and evaluated the map against the design-science criteria of utility, coherence, and actionability [31]; the discovery forum then provided an independent practitioner check on the prioritized subset, while broader external validation remains a limitation noted in Section 6.3. We built the map before the forum and did not share it with participants, so the practitioner reading in Section 4.3 is independent of it. Because the map was developed within a university venture-studio environment, its framing may carry that setting’s commercial orientation; we limited this influence by grounding each SOA in the systematic-review themes and by keeping the map hidden from forum participants, and we return to the residual risk in Section 6.3.

**Table 1:** Derivation of the fifteen meta-jobs from the five themes.

Theme	Meta-Jobs Derived from the Theme (Three per Theme)
Food quality, nutrition, and health outcomes	Deliver food that supports optimal health outcomes; translate nutritional science into scalable innovation; improve transparency and traceability of nutritional value from farm to fork
Sustainable agriculture and food systems	Align agrifood transformation with health and sustainability outcomes; advance resilient and regenerative practices that preserve ecosystem and human health; embed sustainability metrics into decision-making across actors
Community and systemic factors in food access and health	Transform supply chains to equitably serve diverse communities; build multi-stakeholder coalitions to address barriers to nutritious food access; design policy-aligned agrifood innovations for underserved populations
Food as medicine and nutritional interventions	Integrate agrifood innovation into healthcare and preventive nutrition; create scalable supply systems for medically supportive foods; mobilize cost-effective, evidence-based nutrition solutions
Consumer behavior and perceptions	Support informed consumer decisions through transparent practices; respond to evolving consumer values around health and quality; leverage consumer insights to guide health-oriented innovation

### 3.3. An Independent Practitioner Reading: The Discovery Forum

The discovery forum gave us a reading of the opportunity space defined by practitioners on their own terms, with no exposure to the map. It was an invitation-only, two-day event held at Purdue University in October 2025, attended by 37 senior agrifood professionals drawn from across the value chain. Participants were recruited by invitation through the university’s agrifood industry network and partner organizations, targeting senior professionals with strategic responsibility in their firms. Participants spanned input manufacturing, agricultural production, cooperatives and grain handling, food manufacturing, agricultural finance, early-stage ventures, and industry associations; we provide an aggregate, de-identified demographic profile in the Supplementary Materials.

The forum operated at two levels. The full group took part in plenary sessions, including a keynote and a practitioner panel on each day, one panel of food manufacturers and one of agricultural producers, that surfaced system-level dynamics visible only when actors from different segments share a room. At the focused level, seven expert practitioners served as focal informants in six structured ninety-minute discovery sessions in which rotating subgroups conducted Jobs-to-Be-Done interviews and recorded the focal practitioners’ jobs, pains, and gains on Value Proposition Canvas boards [53], following a customer-inquiry protocol designed to elicit genuine needs rather than reactions to a researcher’s framing [54]. We drew the focal panelists deliberately from the two primary segments, four food manufacturing executives and three agricultural producers, because value origination concentrates there and the underlying jobs are expressed most directly at their source. The four-and-three composition followed the availability of focal panelists across the two primary segments rather than a predetermined quota. Actors from the supporting segments took part in the plenary sessions, where their needs entered the discussion as the demand the primary segments transmit to them. Because the focal panel sat entirely within the two primary segments, the discovery could not independently test the primary-supporting distinction; that distinction rests on the literature (Section 2.3), and the forum supplies depth on prioritization within the primary segments, a limitation we return to in Section 6.3. We audio-recorded and transcribed all sessions and coded the canvases and transcripts inductively using the same procedure as the review [49]. The full session protocol is in the Supplementary Materials (S6). Because the forum was a naturalistic discovery exercise rather than a controlled qualitative study, it identifies which opportunities practitioners surface as most pressing, not how frequently each arises across the industry. Recurrence of the same jobs, pains, and gains across the rotating subgroups and across the six sessions is consistent with saturation on the cross-segment needs, although the small panel means saturation cannot be claimed for segment-specific or supporting-segment needs.

### 3.4. Bringing the Views Together: Convergence Analysis

We compared the literature-grounded map with the practitioner-grounded landscape, the step that produces our central empirical result. We assigned each job, pain, and gain articulated in the six focal sessions to one or more SOA coordinates by matching its functional content to the opportunity definition and underlying jobs recorded for each candidate SOA. The lead author performed this assignment against the pre-recorded opportunity definitions and cross-checked it against the plenary evidence; because a single analyst carried out the assignment, we treat it as a structured but not independently replicated coding and note this in Section 6.3. Worked examples of how individual jobs, pains, and gains were assigned to SOAs are given in Supplementary S10. We then computed a convergence score for every SOA as the number of focal sessions, out of six, in which it was referenced, and treated an SOA as high-convergence when it recurred in a majority of the six sessions (at least four) or surfaced independently in both primary segments, a rule that favors opportunities recurring across rotating subgroups over those raised in a single session. The convergence score counts how often an opportunity recurred across the focal sessions; it is not a measure of market size, revenue potential, or commercial importance. We checked that this cutoff does not drive the result by recomputing the set at stricter thresholds and confirming that a lower threshold could only enlarge it (Section 4.4). We cross-checked the high-convergence set against the plenary evidence to corroborate it and to identify opportunities that the focal sessions alone would not reveal, and we identified the structural patterns reported with the results by examining how the high-convergence set distributes across segments, meta-jobs, and themes.

### 3.5. Assessing AI Applicability: Capability Mapping Against the Literature

In the final step we assessed how far current AI can address the prioritized opportunities. For each of the twelve highest-convergence SOAs, we matched the functional requirement implied by the opportunity definition to the AI capability family or families capable of addressing it, drawing on the five families introduced in Section 2.4. We then identified peer-reviewed evidence for each match through targeted searches of the food-science, agricultural, biomedical, and computing literatures, giving preference to recent review and systematic-review articles in established journals. This assessment is a structured but deliberately non-exhaustive scoping of the review literature, proportionate to its role as an applicability overlay on the prioritized opportunities rather than a second systematic review. We verified every reference used in the assessment against the Crossref and Europe PMC metadata records for the canonical publisher version, and we excluded non-peer-reviewed sources, including preprints and trade material, from the evidence base. We graded each opportunity on three explicit criteria: the number of independent peer-reviewed reviews supporting the match, whether those reviews reported validated methods rather than conceptual potential, and whether they documented material reliability limits. We graded an opportunity strong when at least two independent reviews documented validated methods with few reliability caveats, moderate or caveated when the literature was active but reported material reliability limits or rested largely on conceptual demonstrations, and not primarily AI-addressable when the binding constraint was relational, contractual, or market-structural rather than computational. A required step of experimental or clinical validation that follows a technically mature method does not by itself lower a grade; grades were lowered only where the peer-reviewed evidence documented reliability failures in the AI method itself, such as hallucination or unreliable risk-of-bias assessment. A strong grade therefore denotes the maturity of the method under study conditions and does not by itself establish the commercial readiness of a deployed product, which depends on physical testing and integration. The AI-literature search and screening procedure, the inclusion rule, and the per-opportunity application of these criteria are in the Supplementary Materials (S8). A per-opportunity evidence table giving the supporting literature, the validation evidence, the reliability limits, and the rationale for each grade appears as Table S10. This classification is how we distinguish opportunities that AI can address now from those that require other instruments.

## 4. Results: Where Opportunity Concentrates

Our findings build from the problem space toward the prioritized opportunity set. The five themes (Section 4.1) describe the problem space and answer our first question. The structural reading of the map (Section 4.2) establishes where value origination concentrates and characterizes the opportunities as proactive or reactive. The practitioner landscape (Section 4.3) answers the second question by reporting what the industry defines as most pressing. The convergence analysis (Section 4.4) answers the third by identifying where the two views agree and what patterns govern the result. Section 5 then answers the fourth, assessing how AI can address the prioritized set.

### 4.1. The Problem Space: Five Themes

The 34 studies sort into five themes that together describe how food-as-health value arises and where it is constrained (Table 2). The most prevalent, food quality, nutrition, and health outcomes, documents how nutritional composition shapes long-term health and how production and processing decisions mediate that relationship, with biofortification, nutrient-dense varieties, and valorization of byproducts recurring as scalable levers [19,20,22,55]. The strategic reading is that nutritional value is not fixed but is determined by upstream and midstream choices, which creates differentiation potential for firms that can credibly improve it. The second theme, sustainable agriculture and food systems, connects agricultural sustainability to human health through soil, microbiome, and regenerative pathways, and supplies the planetary-health frame that positions production practice as a health lever rather than only an environmental one [16,17,56,57]. The third, community and systemic factors in food access and health, establishes that producing nutritious food is insufficient if it does not reach the populations at stake, and locates much of the action in the channels through which food is delivered, including produce prescription programs and institutional procurement [5,23,24,58].

The fourth theme, food as medicine and nutritional interventions, concerns the explicit use of food to prevent or manage illness, and is the most institutionally developed; it documents the formalization of the Food is Medicine framework, the consensus on how to scale it, and the conditions, namely targeting, payer engagement, and standardized outcome measurement, under which such programs achieve a return [4,28,47]. The fifth, consumer behavior and perceptions, documents persistent gaps between perceived and actual healthfulness and, in its most consequential recent strand, the measurable shift in household purchasing associated with GLP-1 adoption [7,8,25,26]. Read together, the themes locate the origination of food-as-health value in production and manufacturing decisions, with access, intervention, and consumer response shaping how that value is delivered and rewarded.

### 4.2. Where Value Originates, and the Character of Opportunity

The themes do not distribute evenly across the chain, and their distribution bears out the primary-segment lens introduced in Section 2.3. The first two themes implicate agricultural production decisions, including variety selection, agronomic practice, regenerative transition, and on-farm measurement, as the upstream lever that sets the nutritional potential of food before any downstream step. The fourth and fifth themes implicate food manufacturing decisions, including reformulation, functional ingredient sourcing, claim substantiation, healthcare partnership, and consumer communication, as the mechanism through which food-as-health value is delivered and rewarded. The third theme is cross-cutting but operates primarily through manufacturing-facing channels. Across the corpus, the remaining four segments do not appear as independent originators of food-as-health activity; where they appear, their role responds to requirements set elsewhere. On this evidence, we read agricultural production and food manufacturing as the primary segments of the food-as-health value chain, primary in Porter’s [32] sense and the job executors in Ulwick’s [30], while the other four are supporting.

**Figure 2:**
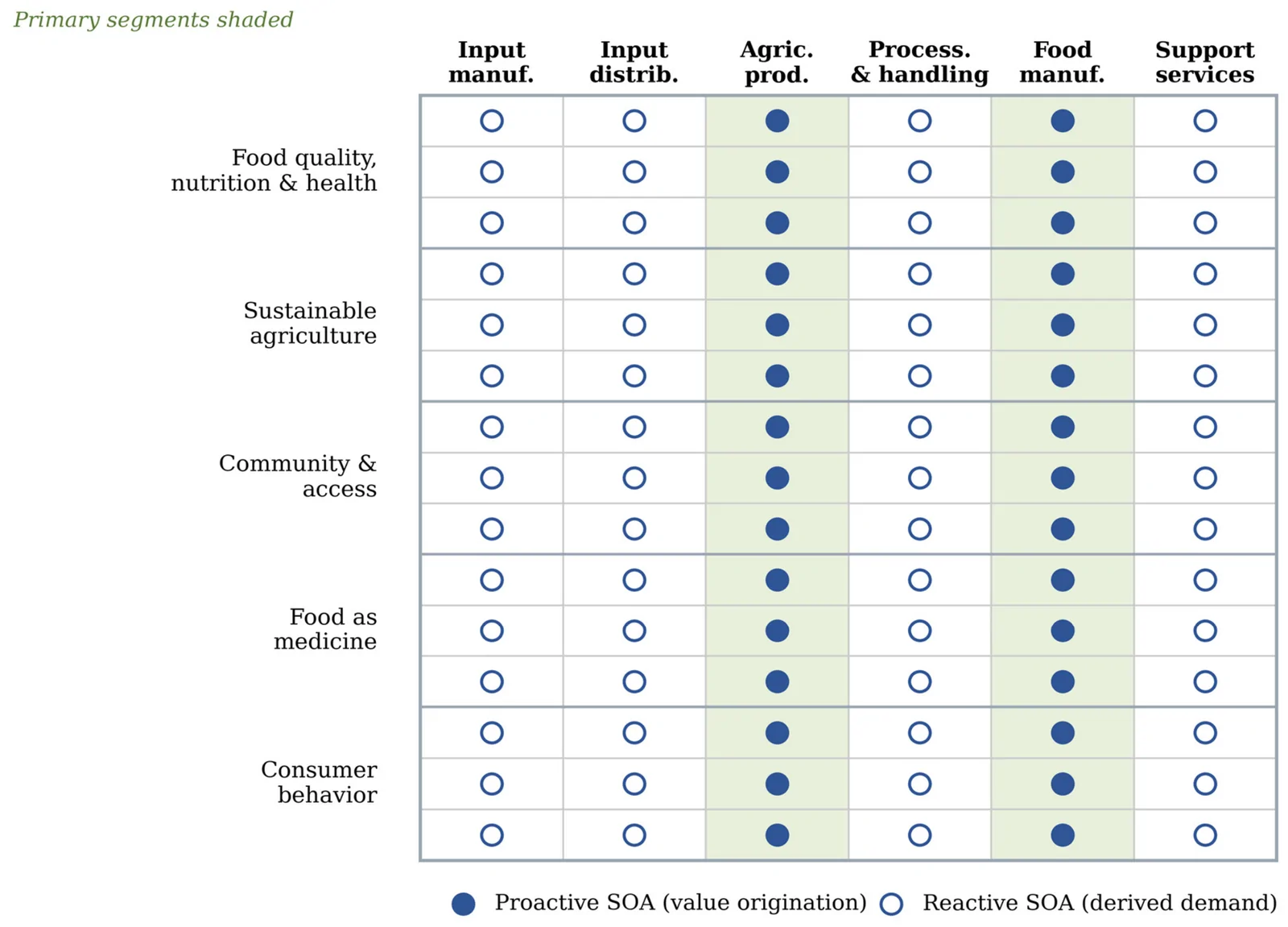
The opportunity map. Fifteen meta-jobs (grouped by theme) cross six value chain segments to define ninety Strategic Opportunity Areas. Shaded columns are the primary segments; filled markers denote proactive SOAs (value origination) and open markers denote reactive SOAs (enabling or derived demand).

This structural reading clarifies what the map does and does not show. Because every meta-job is expressed in every segment, the map contains an opportunity in all ninety cells; it is not denser in some columns than others. The meaningful variation is not in where opportunities exist but in their character. In the two primary segments, opportunities are predominantly proactive: the firm originates health value through its own production or formulation decisions. In the four supporting segments, opportunities are predominantly reactive: they take their shape from what the primary segments require, such as inputs engineered for downstream nutritional density, handling that preserves it, or services that verify it. Figure 2 represents the map on these terms, distinguishing the primary segments and marking each SOA as proactive or reactive. Opportunity originates in the primary segments and propagates outward as derived demand, so that the priority opportunities in the supporting segments are best read as responses to primary-segment needs.

### 4.3. The Practitioner Landscape

The focal sessions produced an independent reading of the opportunity space. Because we drew the focal panelists from the two primary segments, we report the findings by segment, beginning upstream with agricultural production and moving downstream to food manufacturing. Across both, the recurring story is less about the absence of technical solutions than about the absence of the market and information infrastructure that would let firms create and capture health-attributed value.

For agricultural producers, the dominant jobs centered on producing verifiable health-attributed crops or livestock, transitioning toward practices that are simultaneously productive, profitable, and health-attributable, documenting farm-level attributes in buyer-recognized formats, and accessing premium channels, all evaluated against the near-term return that commodity margin volatility demands. The dominant pains formed a coherent pattern: there is no stable price premium for health-attributed commodities, the market infrastructure to transact them is fragmented, the protocols to measure on-farm attributes are absent, and the result is a persistent disconnect between where value is created and where it is captured. The data infrastructure to document attributes is itself a binding constraint, as one operator of a diversified Midwestern operation put it in describing the gap: “I would love nothing more than an SAP system for my operation. Every industry has it. Jiffy Lube has it. I don’t have one for ag.” The remark matters because verification of health attributes presupposes exactly the integrated record-keeping that producers report they lack. The gains producers sought followed directly: a stable premium for verified attributes, integrated decision-support tools, third-party verification, and, above all, long-term contracts with co-investing buyers.

For food manufacturers, the dominant jobs were continuous reformulation under regulatory and consumer pressure, regulatory intelligence as an ongoing operational function rather than an episodic task, strategic rather than transactional supplier collaboration, evidence-based claim substantiation, and portfolio prioritization. The scale of the regulatory burden was concrete: one senior research and development leader at a frozen and refrigerated food manufacturer described more than a thousand products on a multi-tier clean-label transition pathway, characterizing it as “an ongoing journey, because the rules change,” and reported five or six full-time roles devoted to monitoring state-level, customer-specific, and segment-specific requirements manually, in the absence of any centralized system. The dominant pains were consumer skepticism toward branded health claims, regulatory uncertainty across claim categories, supply chain opacity, near-term margin pressure in tension with long-cycle research investment, and the absence of an internal framework for prioritizing among health-oriented initiatives. The gains sought were validated clinical evidence, real-time supply chain traceability, predictable regulatory pathways, specification-grade supplier relationships, and integration with the healthcare system. Table 3 consolidates the focal findings by segment.

Two findings from the focal sessions deserve emphasis. The first is that the same constraint appears on both sides of the chain in mirror image. Producers will invest in health-attributed production if they can secure durable demand, as one operator of a food-grade grain and seed operation explained: “you’d say, well, I’ll build a new plant, but I need a ten-year contract or something. And they’d say, that’s a beautiful story. No thanks.” Manufacturers face the same problem from the other end, where annual best-price pressure works against the long commitments that upstream investment requires: as a sustainability and partnerships leader at a global confectionery company described it, “there’s a lot of pressure in the system to always get the best every year. Some of this requires you’re going to be in bed together for a while.” The symmetry is the point. Both segments want the same instrument, a multi-year commitment that lets value-creating investment be financed, and neither can supply it unilaterally. This is a coordination failure in the governance of the chain rather than a technical gap in any one firm, and it recurs in the discussion as a governance implication.

The second is that the focal evidence converges on a small number of cross-segment needs rather than a long catalog of segment-specific ones. Four needs surfaced consistently in both primary segments: traceability and verification of nutritional value from farm to finished product, market mechanisms that allow health-attributed commodities to be priced and transacted, evidence and substantiation that withstands regulatory and consumer scrutiny, and navigation of a fragmented regulatory environment. That these surfaced independently on both sides of the chain is the first sign of convergence, which the convergence analysis develops formally. The plenary discussions corroborated and extended the focal findings: ingredient sourcing quality and traceability was named by all four manufacturing panelists as a first-order constraint, establishing, in a room that included producers, that the upstream segment must move first for downstream ambitions to be realized; consumer trust emerged as a ceiling observed chiefly from the manufacturing vantage; and regulatory fragmentation was described as a shared cost that propagates upstream through specification changes. These plenary observations are reported as contextual corroboration and were not independently coded.

### 4.4. Convergence and Structural Patterns

Matching the focal evidence to map coordinates identifies the twelve highest-convergence SOAs (Table 4), the opportunities at which the literature-grounded map and the practitioner-grounded landscape agree most strongly. These are our prioritized opportunity set: the opportunities most strongly supported by both the literature and practitioner priorities. We also varied the cutoff to confirm it does not drive the result. Among the twelve, five were referenced in at least five of the six sessions and two in all six, and these higher-convergence opportunities lie entirely in the two primary segments, so tightening the rule sharpens the concentration rather than dispersing it. Lowering the rule below four would only enlarge the set and so cannot displace the higher-convergence opportunities that define the structural reading. The concentration in agricultural production and food manufacturing is therefore invariant to the threshold. Their distribution is not random, and five structural patterns describe it. First, the prioritized opportunities concentrate in the primary segments. Nine of the twelve sit in agricultural production and food manufacturing, and the three in supporting segments are precisely those where derived demand concentrates. This is the distribution that the primary-supporting structure of Section 4.2 anticipates: opportunity in food as health is value-origination-led, most pressing where firms physically determine the health attributes of food, and reaching the supporting segments as the demand the primary segments transmit. Second, propagation as derived demand: the supporting-segment opportunities are the same-meta-job counterparts of primary-segment needs, with traceability the clearest case. Third, cross-segment platform opportunities: four needs converge across both primary segments and are candidates for platform businesses that operate across the chain rather than within a single segment, namely transparency and traceability from farm to fork, evidence and claim substantiation, market infrastructure for health-attributed commodities, and cross-jurisdictional regulatory intelligence. Fourth, a maturity gradient across themes: convergence concentrates in the first, second, and fourth themes, while consumer-oriented needs are visible to manufacturers as a system-level condition but are not yet articulated as discrete, fundable opportunities. Fifth, underweighted opportunities: several opportunities the literature supports drew little practitioner attention, including inputs engineered for downstream nutritional density, nutrient-preservation analytics in processing, and standardized health-attribute audit protocols, marking them as a watchlist for firms willing to move ahead of demand.

## 5. How Artificial Intelligence Can Address the Prioritized Opportunities

**Figure 3:**
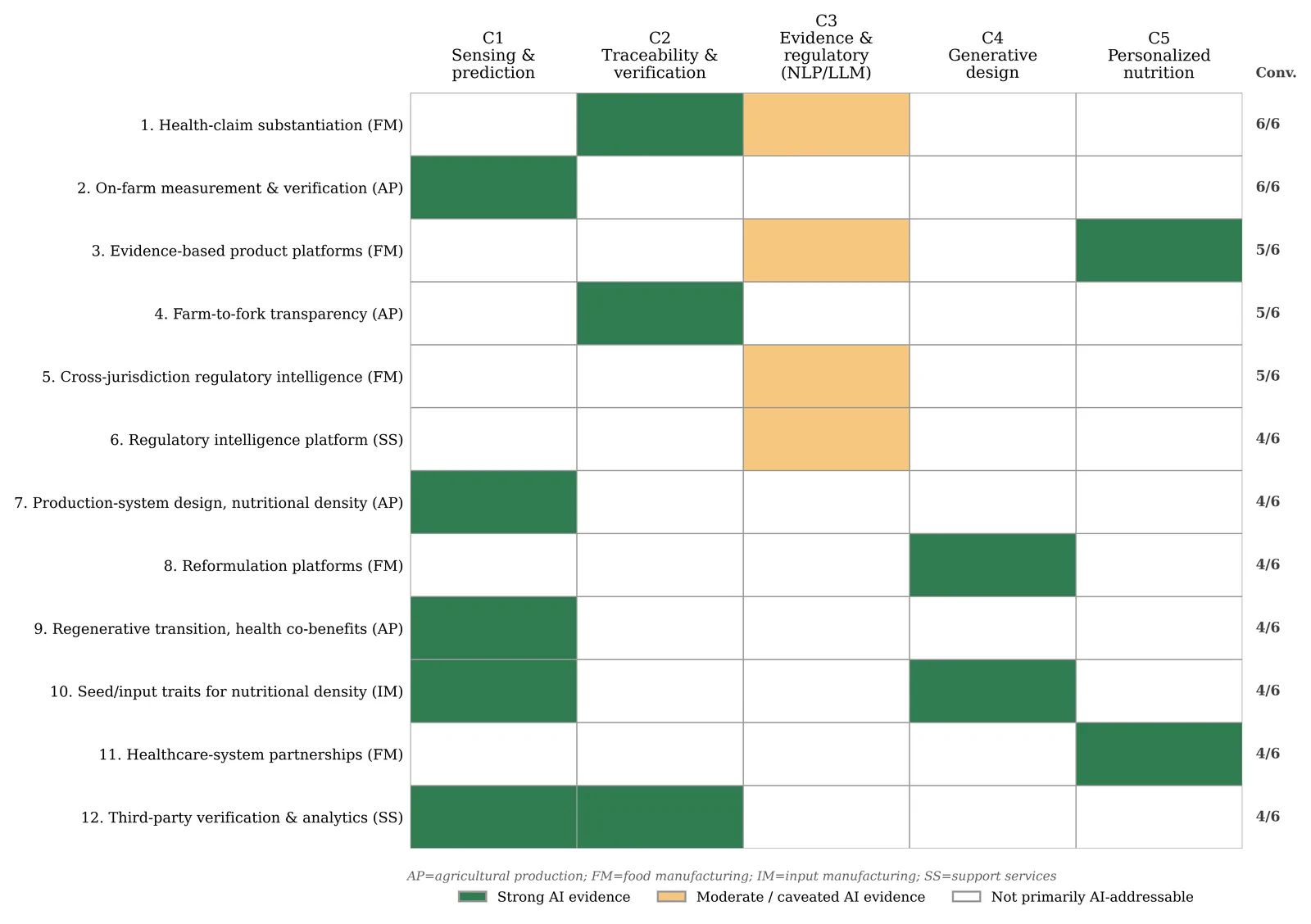
The twelve prioritized opportunities against five AI capability families, shaded by the strength of the peer-reviewed evidence and annotated with the convergence score. The most strongly validated opportunities map onto mature AI capabilities, while the evidence- and regulation-oriented opportunities depend on capabilities with documented reliability limits.

The twelve prioritized opportunities specify what the agrifood industry most needs in the food-as-health space. We now assess how far current artificial intelligence can address them, mapping each opportunity to the AI capability family that applies and grading the strength of the peer-reviewed evidence. The opportunities resolve into five capability families, summarized in Table 5 and Figure 3, and a residual that is not primarily AI-addressable. We are deliberately conservative: we report where mature, validated methods exist, where the literature is active but limited, and where the binding constraint lies outside what computation can resolve.

### 5.1. Sensing, Measurement, and Prediction

The best-supported AI applications address the upstream opportunities of on-farm measurement, production-system design, regenerative transition, and trait development (SOAs 2, 7, 9, and 10). The producer pain that anchored the forum, the absence of protocols to measure on-farm nutritional and health attributes, maps directly onto a mature body of work in which machine learning applied to visible, near-infrared, and hyperspectral data predicts nutritional and quality attributes non-destructively and at scale [35,36]. At the field level, the integration of unmanned aerial vehicles, satellite remote sensing, and machine learning supports the spatially explicit management of nutrient status and crop quality that production-system design for nutritional density requires [37], and remote-sensing models combined with machine learning provide the measurement, reporting, and verification backbone for documenting the health and environmental co-benefits of regenerative transition [60]. Upstream of the field, genomic selection and model-assisted breeding accelerate the development of biofortified and nutritionally improved varieties, addressing the input-side opportunity of designing traits for downstream nutritional density [38,39]. The evidence here is strong: multiple recent reviews document validated methods, and the binding constraint is adoption and integration rather than technical feasibility. This is the clearest case in which AI can supply the measurement infrastructure whose absence the practitioners identified as decisive.

Methodologically, these applications rest on established model classes rather than emerging ones, which is what supports the strong grade. Nutrient and quality estimation from visible, near-infrared, and hyperspectral data typically combines partial least squares regression and support vector machines with convolutional neural networks, and recent reviews report predictive accuracy that is strong for some constituents but highly analyte- and matrix-dependent, with coefficients of determination ranging from moderate to above 0.9 for favorable constituents under good calibration conditions and lower for others [35,36]. Field-scale nutrient and quality management draws on random forests and deep learning applied to multispectral imagery from unmanned aerial vehicles and satellites [37], and soil-carbon monitoring for regenerative claims uses gradient-boosted methods, with reported test coefficients of determination that reach roughly 0.9 in favorable conservation-agriculture settings but vary with soil type and sampling depth [60]. Trait development relies on genomic selection, in which genomic estimated breeding values are computed with Bayesian and machine-learning regression [38,39]. The practical constraint is data rather than algorithms: each method needs calibrated reference libraries and ground-truth measurements, and model transfer across crops, regions, and instruments is limited, so the distance between demonstrated accuracy and routine on-farm use is one of calibration and data infrastructure.

### 5.2. Traceability and Verification

The opportunities for farm-to-fork transparency and third-party verification, and the traceability component of health-claim substantiation (SOAs 4, 12, and 1), map onto a well-developed literature integrating artificial intelligence with the Internet of Things and distributed-ledger technologies. Recent reviews document end-to-end provenance tracking, authentication, fraud detection, and predictive food-safety risk assessment as established applications [13,14,40]. The forum located a bilateral traceability gap, with producers unable to document attributes and manufacturers unable to verify them, and these systems address precisely that gap by making attribute claims legible and auditable across organizational boundaries. The evidence is strong for the technical capability. The qualification is that traceability systems verify what has been measured and recorded; their value therefore depends on the upstream sensing capability of Section 5.1 and on the willingness of chain partners to share data, which returns to the governance question rather than the computational one.

Architecturally, these systems pair distributed-ledger records with machine-learning analytics and Internet-of-Things sensor streams: the ledger provides tamper-evident provenance while supervised models perform authentication, adulteration and fraud detection, and predictive food-safety risk scoring on the captured data [13,14,40]. Reviews describe these as among the more deployment-ready food-AI applications, with commercial pilots already operating, while identifying interoperability and data governance, not algorithms, as the limiting factors, since a ledger verifies only the integrity of what was entered and depends on standardized data exchange among firms that are often reluctant to share it.

### 5.3. Evidence Synthesis and Regulatory Intelligence

The opportunities for health-claim substantiation, cross-jurisdictional regulatory intelligence, and the evidence layer of product platforms (SOAs 1, 3, 5, and 6) map onto natural language processing and large language models, and this is the family where the evidence is most actively contested. On the evidence side, large language models can accelerate biomedical literature search, screening, and data extraction, with reported reductions in screening workload and high extraction accuracy in favorable conditions [42,43]. On the regulatory side, language models can parse regulatory text, extract obligations, and support compliance monitoring across jurisdictions [59], which speaks directly to the manufacturer who described five or six full-time roles monitoring requirements manually. The qualification is substantial and must be stated plainly: the same literature documents hallucination, unreliable risk-of-bias assessment, and limited generalization, and concludes that fully validated applications are not yet established [41,42]. For health-claim substantiation, where the cost of an unsupported claim is regulatory and reputational, these limits mean that AI can augment but not replace expert review, and any deployment requires the kind of verification protocol that responsible practice already demands. The evidence is therefore classified as moderate and caveated rather than strong.

The quantified performance reported for these methods is the reason for the cautious grade. Reviews of large language models in evidence synthesis report substantial reductions in screening workload and data-extraction accuracy in roughly the 80 to 95 percent range under favorable conditions, alongside only slight-to-moderate agreement on risk-of-bias assessment and fabricated references in a non-trivial share of unverified outputs [42,43]. For regulatory text, transformer-based models support clause extraction, obligation identification, and cross-jurisdictional question answering [59]. The implication for health-claim substantiation is specific: these systems suit first-pass retrieval and triage under expert supervision, but the documented hallucination and reliability limits make unsupervised use unsafe where a claim carries regulatory and reputational exposure, which is why the family is graded moderate rather than strong [41].

### 5.4. Generative and Predictive Design

The reformulation opportunity, and the design component of trait development (SOAs 8 and 10), map onto machine learning for formulation, ingredient substitution, and product design. Recent reviews document machine-learning-driven prediction of functional, sensory, and rheological properties that allows reformulation to be explored in silico before physical prototyping, compressing development cycles [9,12], and a dedicated review frames AI-enabled ingredient substitution across sensory, functional, nutritional, and cultural dimensions [45]. The manufacturer who described more than a thousand products on a clean-label transition pathway is describing exactly the combinatorial reformulation problem that these methods address. Beyond incremental reformulation, machine learning supports the development of novel and alternative-protein products, including the optimization of culture media for cultivated meat [44], an application squarely within this Special Issue’s scope. The evidence is strong for the predictive capability, with the standing caveat that model outputs require experimental validation before they reach the market.

The underlying methods span supervised property prediction and generative design. Machine-learning models estimate functional, sensory, and rheological properties from formulation composition, allowing candidate reformulations to be ranked in silico before bench work, while generative and optimization approaches propose ingredient substitutions and new formulations [9,12,45]. In the alternative-protein domain, the same machinery optimizes culture-media composition and bioprocess parameters for cultivated meat [44]. Reviews report compressed development cycles as the principal demonstrated benefit; the standing limit is that model predictions narrow the experimental search space rather than replace it, so laboratory validation remains a required step before any reformulation reaches the market.

### 5.5. Personalized Nutrition and Decision Support

The opportunity for healthcare-system partnerships, and the clinical-integration component of evidence-based platforms (SOAs 11 and 3), map onto AI for personalized nutrition and clinical decision support. Recent reviews document the translation of individual biological and behavioral data into dietary recommendations, the use of continuous glucose monitoring with machine learning for glycemic decision support, and outcome prediction relevant to the targeting that Food is Medicine programs require to achieve a return [10,11,46]. This is the capability that connects the food-as-medicine theme to a reimbursable business model, because payer engagement depends on demonstrating outcomes for defined populations, which is precisely what these methods support. The evidence is strong and growing, with the qualification that clinical deployment carries regulatory and validation requirements beyond those of the other families.

These applications draw on supervised learning and, increasingly, on deep and federated learning applied to continuous glucose monitoring, dietary intake, and behavioral and multi-omic data, producing individualized recommendations and outcome predictions [10,11,46]. For the food-as-medicine opportunity, the operative capability is outcome prediction that supports population targeting and demonstrates effect for defined groups, which is the evidentiary basis payers require for reimbursement. The constraint is regulatory rather than computational: clinical deployment requires validation and, depending on the claims made, regulatory clearance beyond what the other capability families face.

### 5.6. The Boundary: What AI Cannot Address

Our assessment also marks a boundary. The single most consequential finding of the forum, the symmetric long-term-contract gap, is not an AI problem. Both primary segments seek a durable multi-year commitment that would let value-creating investment be financed, and neither can supply it unilaterally. This is a coordination failure in the governance of the chain, and no sensing model, traceability ledger, or language model resolves it. AI can lower the information costs that make such commitments hard to write, by making attributes measurable and verifiable and by clarifying regulatory exposure, but the commitment itself is relational and contractual. The same boundary applies to the market mechanisms for pricing health-attributed commodities that surfaced as a cross-segment need: AI can supply the verified attribute data on which a premium market would depend, but it cannot create the market. Recognizing this boundary protects against the diffuse over-claiming that characterizes much of the AI-in-food discourse and locates AI accurately as the enabling layer for measurement, verification, evidence, design, and personalization, within a value-creation problem whose binding constraint is governance.

Three cross-cutting considerations further bound what AI contributes here. Model performance is limited by the representativeness of the training and calibration data, so systems built on narrow or non-representative data can encode and propagate bias, a limit the reliability literature documents most sharply for the evidence-synthesis family [42]. The data, calibration, and computation these methods require also fall unevenly across the chain, favoring larger and better-resourced firms over smaller producers and raising a distributional question about who captures AI-enabled health value. And the infrastructure and energy footprint of large models is itself a sustainability cost that a food-systems deployment should weigh rather than assume away.

## 6. Discussion

### 6.1. Where Value Originates, and What Current AI Can Address

Our analysis yields one main result and one methodological one. The main result concerns where value originates in the food-as-health chain [15,18]. Food-as-health innovation is value-origination-led, concentrated in the two segments that physically determine the health attributes of food and propagating outward to the remaining segments as derived demand. The systematic review, read through value chain and Jobs-to-Be-Done theory, establishes this structure, and the forum shows it in operation: practitioner priorities fall where the structure locates value, and the constraint that governs them is the one the discussion makes visible. The reading runs against a plausible alternative in which input manufacturing, where seed traits and functional ingredients are designed, would drive the field; the literature indicates that its food-as-health role is predominantly demand-derived under our lens, even though technology-push origination also occurs [34]. The symmetric long-term-contract gap sharpens the reading: both primary segments seek the same instrument, a durable commitment that would let value-creating investment be financed, and neither can supply it alone, so the binding constraint on food-as-health value creation is relational and contractual rather than technical. The AI assessment reinforces this from the opposite direction: the opportunities current AI addresses most convincingly are those of measurement, verification, design, and personalization, and the constraint it cannot address is the contractual one, which confirms that the structural lens of value origination and derived demand does explanatory work a purely technological account would miss. The methodological result is that the meta-job construct travels: practitioner-defined jobs sorted cleanly into the meta-job structure we derived independently from the literature, which extends Jobs-to-Be-Done analysis [29] from the single-firm setting to a value chain in which one consumer-defined need is met by many actors at once [31].

### 6.2. Implications for Practice, Investment, and Policy

For food manufacturers, the twelve convergent opportunities define a concentrated agenda. Evidence-based product platforms that integrate clinical research, supply-chain traceability, and healthcare distribution are the most strongly validated territory, and the AI assessment indicates that the measurement, traceability, and personalization components are addressable now, while the evidence-synthesis component requires the human-in-the-loop verification that the reliability literature demands. Reformulation is best organized as a continuous operational capability supported by predictive design tools, and regulatory intelligence, currently performed manually by dedicated teams, is a candidate for language-model-assisted consolidation, subject to the same reliability caveats. For agricultural producers, the central implication is the value-creation-versus-value-capture gap: producers generate upstream nutritional value but rarely capture the premium, and the three convergent production opportunities, on-farm measurement and verification, farm-to-fork transparency, and production-system design for premium capture, are the levers that close it, each now supported by mature sensing and prediction methods. The forum makes plain that the binding constraint is the absence of multi-year buyer commitments, so the decisive move is contractual as much as technical, and AI is best understood as lowering the information cost of writing such commitments rather than substituting for them. For investors and innovators, the four cross-segment platform needs are the opportunities with the broadest base, and the underweighted opportunities are where first movers can act ahead of demand; the AI mapping identifies which of these are technically ready and which await capability maturation. For policymakers, the recurring pattern of buyer–supplier short-termism, immature measurement infrastructure, and uneven technology fit points to a concrete enabling agenda: contracting norms that internalize multi-year horizons, public investment in measurement and verification infrastructure, and applied research that adapts promising AI tools to operational heterogeneity. Because the data, calibration, and computation that these tools require fall unevenly across the chain, this agenda should also keep AI-enabled advantage from concentrating among the largest firms; public or cooperatively held reference libraries, shared calibration and measurement infrastructure, and subsidized access for smaller producers would let upstream measurement gains reach the operations that most need them [61,62].

### 6.3. Limitations and Future Research

Several limitations bound our claims. Our convergence analysis rests on seven focal panelists across six sessions, all from the two primary segments. This supports identification of where opportunity concentrates rather than an estimate of how frequently each opportunity arises across the industry, and because the panels sit within the primary segments, the primary-supporting structure rests on the literature rather than on an independent practitioner test; the supporting segments enter only through plenary corroboration and the derived-demand opportunities the focal sessions surface. The corpus and the practitioner sample are restricted to the United States or a United States-applicable context, so transfer to other regulatory environments is not established, and the AI capabilities assessed, particularly regulatory intelligence, are jurisdiction-sensitive. The priorities identified here should therefore be treated as specific to the United States context until tested elsewhere [63], and Section 7 develops the transferability question. Participants were recruited through university and industry networks, which may favor firms already engaged with the food-as-health agenda. The two coding streams differ in how their reliability can be expressed. For the systematic review, although the original screen used a distributed protocol in which five reviewers screened different databases rather than overlapping records, we quantified screening reliability directly through a post hoc check in which two members of the research team independently re-screened a random sample of 50 records against the inclusion criteria; agreement was moderate (Cohen’s κ = 0.56; see Section 3.1). The workshop coding stream, by contrast, captured jobs, pains, and gains through facilitated consensus across rotating subgroups rather than parallel double-coding; it rests on transparent criteria, a shared instrument, and consensus resolution, an accepted basis for interpretive synthesis [51], and should therefore be read as structured rather than statistically measured. The included corpus is also heterogeneous and weighted toward review and non-empirical sources; a formal appraisal of the empirical subset (Supplementary S9) found generally moderate quality, with recurring limits in sample representativeness and in the completeness of methods reporting, which bounds the strength of the evidence base. Because we built the map from the same review we used to identify the themes, there is a risk of circular validation that the blind forum mitigates but cannot wholly remove. The convergence assignment was carried out by a single analyst, so the mapping of practitioner statements to opportunities is structured but not independently replicated. Finally, the AI assessment is a structured reading of the current peer-reviewed literature rather than an implementation study; it establishes that capabilities exist and how strong the evidence is, not that any given firm can deploy them at an acceptable cost, and the fast-moving nature of the field means the evidence base will shift. Four directions follow: Extending structured discovery to the four supporting segments would test the propagation pattern directly. Applying the value-origination lens to other agrifood innovation domains would test how far the structural finding generalizes. Piloting the best-supported AI applications, on-farm sensing and traceability in particular, would convert the capability assessment into deployment evidence. And tracking whether convergence-based priority rankings predict actual firm investment over time would turn a descriptive prioritization into a predictive one.

## 7. Future Perspectives

The assessment in Section 5 reflects the peer-reviewed evidence available at the time of writing, and that evidence is expanding quickly. Recent systematic reviews document the widening application of machine learning across agricultural production [64], food processing [65], food quality and safety detection [66], and food-security analysis [67,68], and they report both maturing capability and a persistent gap between demonstrated accuracy and field deployment. For the opportunities identified here, the most consequential near-term progress is in the sensing, traceability, and design families, where model performance and data availability continue to improve.

Two emerging paradigms are likely to reshape the landscape. Foundation models, trained on broad data and adapted to specific tasks, are beginning to enter agriculture and food science and may lower the cost of building the measurement and evidence-synthesis tools that several prioritized opportunities require [69,70]. Digital twins and federated learning point toward continuous, privacy-preserving analysis across firm boundaries, which speaks to the traceability and data-sharing constraints the forum identified. These paradigms remain early, and their reliability under operational conditions is not yet established.

Realizing these opportunities depends less on algorithms than on the conditions for deployment. The adoption literature identifies a consistent set of barriers: uneven data quality and availability, limited interoperability across proprietary systems, weak explainability in high-stakes decisions, questions of model robustness and cybersecurity as food systems digitize, regulatory uncertainty, and the cost and skill requirements that slow adoption, particularly for smaller firms [63,71]. These barriers fall unevenly, and firms that already hold data and technical capacity are best placed to capture the gains, which returns to the distributional concern of Section 5.6.

The evidence base and the practitioner panel are focused on the United States, and the applicability of the framework elsewhere is an open question. Production systems, digital infrastructure, and regulatory regimes differ substantially across regions, and in many developing-country settings the connectivity and calibrated measurement that several opportunities presuppose are themselves constrained. Extending the analysis to other regions, and testing which opportunities and AI capabilities transfer, is a priority for future work.

## 8. Conclusions

We set out to identify where the most strongly supported food-as-health opportunities lie for the agrifood industry, why, and how far current artificial intelligence can address them. Combining a systematic review, a Jobs-to-Be-Done opportunity map, and a blind practitioner discovery forum, and bringing them together through a convergence analysis, we find that food-as-health value origination concentrates in two primary segments, agricultural production and food manufacturing, while the remaining segments host reactive opportunities driven by derived demand. Twelve opportunities at which literature and practice converge form a concentrated agenda built around four cross-segment platform needs: traceability and verification, evidence substantiation, market mechanisms for health-attributed commodities, and regulatory navigation. Assessing these against the peer-reviewed literature, we find that current AI addresses them through five capability families, with strong evidence for sensing, traceability, design, and personalization and more qualified evidence where reliability limits require human oversight. The most consequential constraint, the symmetric long-term-contract gap, lies outside what AI can resolve and is relational rather than computational. These conclusions rest on a United States-focused evidence base and a small practitioner panel, and the AI assessment reflects a fast-moving literature, as Section 6.3 details. We offer a prioritized, evidence-grounded map of where to compete, a clear account of which opportunities AI can act on now, and an explicit boundary on its reach. That combination gives firms, investors, and policymakers a defensible basis for deciding where to act and which tools to use.

## Figures and Tables

**Table 2:** The five themes in the food-as-health literature. Theme counts exceed the corpus size because studies could be coded to more than one theme.

Theme	*n*	Core Topics
Food quality, nutrition, and health outcomes	23	Biofortification; nutrient density; functional foods; nutrient preservation
Sustainable agriculture and food systems	16	Regenerative practice; soil and microbiome pathways; nutrition-sensitive agriculture
Community and systemic factors in food access and health	13	Affordability and access; produce prescription programs; procurement coordination
Food as medicine and nutritional interventions	10	Clinical food intervention; medically tailored meals; healthcare integration
Consumer behavior and perceptions	6	Consumer beliefs and trust; GLP-1 demand effects; social media

**Table 3:** Practitioner jobs, pains, and gains from the six focal discovery sessions, by primary segment.

	Agricultural Production (Upstream)	Food Manufacturing (Downstream)
Jobs	Produce verifiable health-attributed crops or livestock; transition to nutritionally oriented practices; document farm-level attributes in buyer-recognized formats; access premium channels; manage yield-nutrition tradeoffs	Reformulate continuously under regulatory and consumer pressure; sustain regulatory intelligence as an operational function; collaborate strategically with suppliers; substantiate clinical evidence; prioritize the portfolio
Pains	No stable price premium; fragmented market infrastructure; absent measurement protocols; value-creation versus value-capture disconnect; regulatory uncertainty on producer claims	Consumer skepticism toward branded claims; regulatory uncertainty across claim categories; supply chain opacity; near-term margin pressure versus long-cycle research; no internal prioritization framework
Gains	Stable premium for verified attributes; long-term contracts with co-investing buyers; integrated decision-support tools; third-party verification	Validated clinical evidence; real-time supply chain traceability; predictable regulatory pathways; specification-grade supplier relationships; healthcare-system integration

**Table 4:** The twelve highest-convergence Strategic Opportunity Areas. The convergence score is the number of focal sessions (of six) in which the SOA was referenced; the final column indicates plenary corroboration. Pr = primary segment; Su = supporting segment.

#	Segment	Strategic Opportunity Area	Score	Plenary
1	Food manufacturing (Pr)	Health-claim substantiation with traceable clinical and supply-chain evidence	6/6	Yes
2	Agricultural production (Pr)	On-farm measurement and verification of nutritional and health attributes	6/6	Yes
3	Food manufacturing (Pr)	Evidence-based product platforms with clinical-trial integration	5/6	Yes
4	Agricultural production (Pr)	Farm-to-fork transparency for health-positioned products	5/6	Yes
5	Food manufacturing (Pr)	Cross-jurisdictional regulatory intelligence for health claims	5/6	Yes
6	Support services (Su)	Regulatory intelligence platform across jurisdictions	4/6	Yes
7	Agricultural production (Pr)	Production-system design for nutritional density and premium capture	4/6	Yes
8	Food manufacturing (Pr)	Reformulation platforms balancing nutrition and perceived quality	4/6	No
9	Agricultural production (Pr)	Regenerative transition with documented health co-benefits	4/6	Yes
10	Input manufacturing (Su)	Seed traits and inputs designed for downstream nutritional density	4/6	No
11	Food manufacturing (Pr)	Healthcare-system partnerships for reimbursable products	4/6	Yes
12	Support services (Su)	Third-party verification and analytics for farm-to-product attributes	4/6	Yes

**Table 5:** Mapping of the twelve prioritized opportunities to current AI capability families and the strength of the peer-reviewed evidence. Capability families: C1 sensing and prediction; C2 traceability and verification; C3 evidence synthesis and regulatory intelligence; C4 generative and predictive design; C5 personalized nutrition and decision support. A strong grade denotes the maturity of the underlying method under study conditions and does not by itself establish commercial readiness, which requires physical testing. Where an opportunity references premium capture or market access (SOA 7), the grade applies to the measurement and design component; the premium and market mechanisms themselves are not AI-addressable (Section 5.6).

#	Prioritized Opportunity	AI Capability Family	Evidence	Representative Literature
1	Health-claim substantiation	Traceability + evidence synthesis (C2, C3)	Moderate	[14,43]
2	On-farm measurement and verification	Sensing and prediction (C1)	Strong	[35,36]
3	Evidence-based product platforms	Evidence synthesis + personalization (C3, C5)	Moderate	[11,42]
4	Farm-to-fork transparency	Traceability and verification (C2)	Strong	[13,40]
5	Cross-jurisdictional regulatory intelligence	Regulatory NLP/LLMs (C3)	Moderate	[41,59]
6	Regulatory intelligence platform	Regulatory NLP/LLMs (C3)	Moderate	[42,59]
7	Production-system design for nutritional density	Sensing and prediction (C1)	Strong	[37,60]
8	Reformulation platforms	Generative and predictive design (C4)	Strong	[12,45]
9	Regenerative transition with health co-benefits	Sensing and dMRV (C1)	Strong	[37,60]
10	Seed/input traits for nutritional density	Sensing + generative design (C1, C4)	Strong	[38,39]
11	Healthcare-system partnerships	Personalized nutrition and decision support (C5)	Strong	[10,46]
12	Third-party verification and analytics	Sensing + traceability (C1, C2)	Strong	[13,36]

## Data Availability

The data supporting the findings of this study, including the opportunity-map documentation and aggregated forum outputs, are contained within the article and the Supplementary Materials. The full discovery-forum transcripts are not publicly available to protect participant confidentiality and may be requested from the corresponding author subject to confidentiality conditions.

## Supplementary Materials

The following supporting information can be downloaded at https://www.mdpi.com/article/10.3390/foods15183215/s1, the full PRISMA 2020 search strings and flow diagram; the characteristics table for the 34 included studies; the inductive coding progression; the complete documentation of the ninety Strategic Opportunity Areas; the aggregate, de-identified participant demographic profile; the discovery-forum facilitator protocol; a methodological quality appraisal of the included studies; and a per-opportunity evidence table for the prioritized opportunities. Table S1. Records identified by source. The seven database searches returned 227 records; targeted citation searching within Google Scholar added the two foundational nutrition-sensitive agriculture studies, for 229 records identified in total. Google Scholar records include work indexed there but not consistently in the bibliographic databases. Figure S1. PRISMA 2020 flow diagram for the systematic review. Table S2. The 34 included studies. Theme codes: T1 food quality, nutrition, and health outcomes; T2 sustainable agriculture and food systems; T3 community and systemic factors in food access and health; T4 food as medicine and nutritional interventions; T5 consumer behavior and perceptions. Table S3. Final themes and member studies (by # in Table S2). Figure S2. Inductive coding progression from 96 initial codes through 8 intermediate categories to the five final themes. Edges indicate the theme or themes to which each category’s studies were assigned; studies could be assigned to more than one theme. Table S4. The fifteen meta-jobs by theme. Table S5. Bounded opportunity definition for the ninety Strategic Opportunity Areas, organized by meta-job (SOA 01 to 90). Table S6. Participants by sector represented (n = 37). Classification is approximate and based on organization type. Table S7. Participants by role and seniority (n = 37). Classification is approximate and based on job title. Table S8. The five AI capability families, the prioritized SOAs each addresses, the strength of the peer-reviewed evidence, and the principal reliability caveat. The long-term-contract and market-creation constraints identified in the forum are not addressable by any family (Section 5.6). Table S9. Methodological quality appraisal of the 34 included studies. Empirical studies were appraised with MMAT 2018; reviews with a review-appropriate check; non-empirical items were classified and retained as contextual evidence, not formally scored. Table S10. Per-opportunity evidence base for the twelve prioritized opportunities. Capability families: C1 sensing, measurement, and prediction; C2 traceability and verification; C3 evidence synthesis and regulatory intelligence; C4 generative and predictive design; C5 personalized nutrition and decision support. A strong grade denotes method maturity under study conditions and does not by itself establish commercial readiness, which requires physical testing.
