# Supplementary material for: Prioritizing Artificial Intelligence Opportunities for Food as Health in the U.S. Agrifood Value Chain
Source: Foods. 2026 Sep 11;15(18):3215. doi: 10.3390/foods15183215 (PMC13606927; doi:10.3390/foods15183215)
Supplement: Supplementary file 1 [file foods-15-03215-s001.zip › foods-4490493-supplementary.pdf]

Supplementary Materials

Prioritizing Artificial Intelligence Opportunities for Food as Health in the U.S. Agrifood Value Chain

Lourival Carmo Monaco Neto 1,* and Allan W. Gray 1
1 Department of Agricultural Economics, College of Agriculture, Purdue University, West Lafayette, IN 47907, USA
* Correspondence: lmonacon@purdue.edu

This document provides the procedural detail supporting the instruments described in the main text: the systematic review (S1 to S3), the opportunity map (S4), the practitioner discovery forum (S5 to S7), the AI capability assessment (S8), the methodological quality appraisal (S9), and the per-opportunity evidence base (S10). It is referenced from the main manuscript and is not intended to be read independently of it. All forum material is reported in aggregate and de-identified; no participant or company is named.

S1. Search Strategy and Identification

The review followed the PRISMA 2020 reporting guidelines. Seven databases were searched for English-language work published between January 2015 and May 2025: PubMed, Scopus, Web of Science, Google Scholar, AGRIS, CAB Direct, and Embase. A single Boolean string was applied to every database, with Google Scholar added as a supplementary source to capture high-impact and cross-disciplinary work not consistently indexed by the bibliographic databases.

Boolean search string: ("food as health" OR "food is health" OR "food as medicine") AND ("agricultural practices" OR "food systems" OR "agriculture value chain" OR "agricultural production" OR "agricultural systems" OR "food production" OR "food manufacturing").

The same string was applied to each database, adapted to its native syntax and covering the title, abstract, and keyword fields. Searches were limited to English-language work published between January 2015 and May 2025 and were run between January and May 2025; eligibility by publication type, peer-reviewed studies together with established institutional reports, was applied during screening rather than as a database filter. Because the reviewers screened the databases separately under the distributed protocol, the records were not pooled before screening, and de-duplication was carried out as a post hoc check on the combined identification log after screening was complete. That check identified five duplicate records arising from four works: one work was returned by three databases and three works were returned by two databases each. Because each duplicate had already been screened within its own database, the counts reported in Table S1 and Figure S1 are the log counts and include these records. Two of the four works are among the studies retained for analysis, each represented once, so de-duplication does not change the corpus of 34 studies.

The seven database searches returned 227 records. Targeted citation searching within Google Scholar added two foundational nutrition-sensitive agriculture studies (Willett et al., 2019; Sharma et al., 2021), for 229 records identified in total. After screening, 34 studies were retained for analysis, comprising 32 studies from the database searches and the two studies added through citation searching. Identification by source is reported in Table S1 and the flow is shown in Figure S1.

Screening was conducted by a team of five reviewers under a distributed protocol. Each reviewer was assigned a subset of the databases and screened its records against the inclusion criteria, recording include, exclude, or uncertain decisions in a shared instrument. The team resolved inclusion by consensus, bringing records flagged uncertain and any full-text disagreements to joint discussion. Because the reviewers screened different databases rather than overlapping records, the primary screen produced no overlapping subset. To quantify screening reliability, two members of the research team re-screened a random sample of 50 records as a post hoc check, after the original screening was complete, independently and blind to the original decisions; on the binary advance-versus-exclude decision, agreement was Cohen's $\kappa = 0.56$ (90.0% observed agreement; 95% CI 0.22–0.90). The primary protocol otherwise relied on transparent criteria, the shared decision instrument, and consensus resolution.

Of the 227 database records, 180 were excluded at title and abstract and 47 were assessed at full text. Fifteen were excluded at full text. The recurring grounds were a focus on a non-United States context without demonstrable applicability to it (for example, studies centered on Iran, China, or the Caribbean), an ethnobotanical or biodiversity survey without an agrifood-system framing (for example, inventories of wild or digestive plants), no substantive linkage between the food or agricultural subject and a health outcome (for example, work centered on energy or water rather than nutrition), and introductory or editorial material rather than a study or report. Thirty-two database studies were retained; with the two studies added through citation searching, the corpus comprised 34 studies. Title-and-abstract and full-text decisions, with reasons, are recorded in the project screening log.

Source	Records
Scopus	100
Google Scholar	103
PubMed	15
CAB Direct	3
Web of Science	2
AGRIS	2
Embase	2
Subtotal, database searches	227
Identified outside the database searches	2
Total identified	229
Included after screening	34

Table S1. Records identified by source. The seven database searches returned 227 records; targeted citation searching within Google Scholar added the two foundational nutrition-sensitive agriculture studies, for 229 records identified in total. Google Scholar records include work indexed there but not consistently in the bibliographic databases.

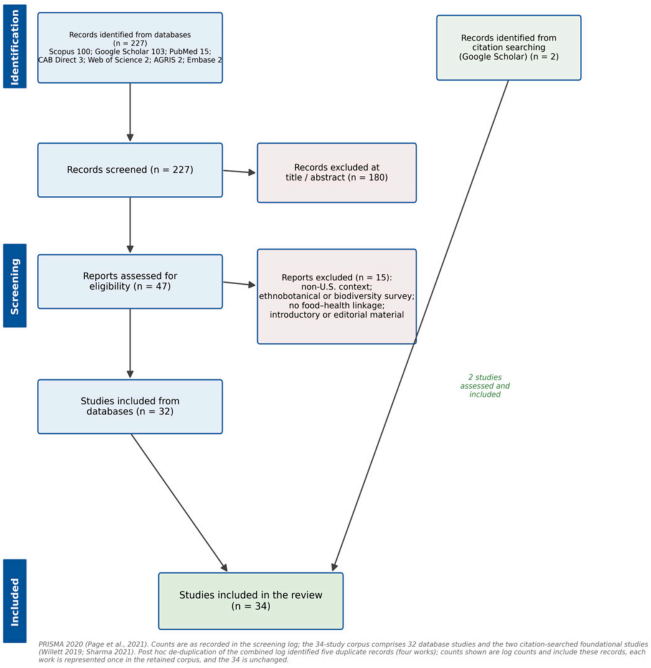

Figure S1. PRISMA 2020 flow diagram for the systematic review.

Funnel counts are drawn from the project screening log: 227 database records identified, 180 excluded at title and abstract, 47 assessed at full text, and 32 studies retained, to which the two studies located through citation searching were added for a final corpus of 34.

S2. Characteristics of the 34 Included Studies

Table S2 lists the 34 studies retained after screening, with first author and year, journal, identifying database, and the theme or themes to which each was coded. Studies could be coded to more than one theme, so theme assignments sum to more than 34. Bibliographic details for studies also cited in the main text follow the verified records in the main reference list; details for studies appearing only in the review are drawn from the project study database.

#	First author (Year)	Journal	Database	Theme(s)
1	Johnson et al. (2023)	Clinical Pediatrics	Scopus	T3,T4
2	McConnell et al. (2016)	Journal of Agricultural and Food Chemistry	PubMed	T1,T2
3	Ylitalo et al. (2024)	Translational Behavioral Medicine	Scopus	T1,T3,T4
4	Li et al. (2024)	Nature Food	Scopus	T1

#	First author (Year)	Journal	Database	Theme(s)
5	Clark et al. (2022)	Nutrition Bulletin	Scopus	T1,T3,T4
6	Guardiola-Márquez et al. (2022)	Plants	Google Scholar	T1,T2
7	Roe (2025)	Choices Magazine	Google Scholar	T1,T5
8	Bernstein et al. (2024)	American Journal of Lifestyle Medicine	Scopus	T2,T4
9	Ridberg et al. (2024)	The American Journal of Clinical Nutrition	Scopus	T1,T3,T4
10	Volpp et al. (2023)	Circulation	Google Scholar	T1,T3,T4
11	Sollid et al. (2022)	Nutrition Today	Scopus	T5
12	Mihafu et al. (2020)	Current Research in Nutrition and Food Science	Google Scholar	T1
13	Rahman et al. (2024)	Frontiers in Nutrition	Scopus	T1,T2,T3,T4
14	Muchenje et al. (2018)	Food Research International	PubMed	T1,T2,T3,T5
15	Maestre et al. (2018)	Institute of Development Studies	Google Scholar	T1,T3
16	Kline et al. (2024)	Local Environment	Scopus	T2,T3,T4
17	Vogl et al. (2016)	Journal of Ethnobiology and Ethnomedicine	Embase	T3
18	Venn (2020)	Nutrients	PubMed	T1
19	Mattoo et al. (2023)	Frontiers in Sustainable Food Systems	Scopus	T1,T2
20	Erisman et al. (2021)	Organic Agriculture	Google Scholar	T1,T2
21	Rahmann et al. (2016)	Organic Agriculture	Google Scholar	T2
22	Ohno et al. (2018)	Journal of Environmental Quality	PubMed	T2
23	Higgins et al. (2022)	Southeastern Geographer	Scopus	T3
24	Shroff et al. (2020)	Development	Google Scholar	T1,T2
25	Hristakeva et al. (2025)	Cornell SC Johnson College of Business	Google Scholar	T1,T5
26	Olson (2017)	Journal of Cleaner Production	Scopus	T1,T2,T5
27	Choe et al. (2018)	Journal of Agricultural and Food Chemistry	Scopus	T1
28	Wadhwa (2020)	Explore	Embase	T3,T4
29	Fatemi et al. (2023)	British Food Journal	Scopus	T1,T5
30	Shi et al. (2023)	Frontiers in Sustainable Food Systems	Scopus	T1,T2
31	Tian et al. (2024)	LWT - Food Science and Technology	Scopus	T1,T2
32	Fredericks et al. (2024)	Journal of General Internal Medicine	Scopus	T1,T3,T4
33	Willett et al. (2019)	The Lancet	Google Scholar	T2
34	Sharma et al. (2021)	Advances in Nutrition	Google Scholar	T2

Table S2. The 34 included studies. Theme codes: T1 food quality, nutrition, and health outcomes; T2 sustainable agriculture and food systems; T3 community and systemic factors in food access and health; T4 food as medicine and nutritional interventions; T5 consumer behavior and perceptions.

Journals for studies appearing only in the systematic review (not cited in the main text) are drawn from the project study database. The two foundational studies added through citation searching, Willett et al. (2019) and Sharma et al. (2021), are classified to Theme 2 (Sustainable Agriculture and Food Systems).

S3. Inductive Coding

Coding followed a general inductive approach (Thomas, 2006) appropriate to a heterogeneous corpus, with synthesis-without-meta-analysis principles guiding the narrative consolidation. The same five reviewers progressed through three rounds. Round 1 generated initial open codes during independent reading (96 codes). Round 2 aggregated the initial codes into intermediate categories through team discussion (8 categories). Round 3 consolidated the intermediate categories, by team consensus, into the five final themes. The progression is shown in Figure S2, and the membership of each final theme is reported in Table S3.

Theme	n	Member studies (by # in Table S2)
T1 Food quality, nutrition, and health outcomes	23	2, 3, 4, 5, 6, 7, 9, 10, 12, 13, 14, 15, 18, 19, 20, 24, 25, 26, 27, 29, 30, 31, 32
T2 Sustainable agriculture and food systems	16	2, 6, 8, 13, 14, 16, 19, 20, 21, 22, 24, 26, 30, 31, 33, 34
T3 Community and systemic factors in food access and health	13	1, 3, 5, 9, 10, 13, 14, 15, 16, 17, 23, 28, 32
T4 Food as medicine and nutritional interventions	10	1, 3, 5, 8, 9, 10, 13, 16, 28, 32
T5 Consumer behavior and perceptions	6	7, 11, 14, 25, 26, 29

Table S3. Final themes and member studies (by # in Table S2).

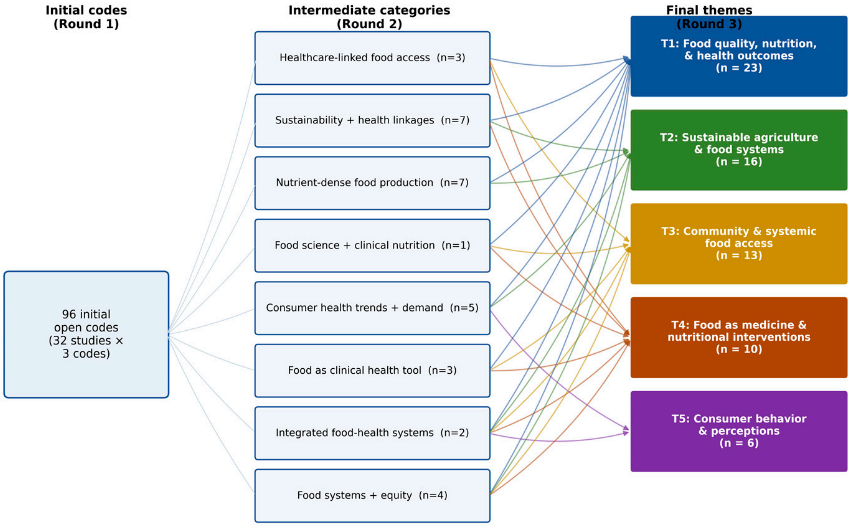

Inductive coding progression (Thomas, 2006): 96 open codes across the 32 systematically screened studies were aggregated by team consensus into 8 intermediate categories and consolidated into the 5 final themes. Edges show the theme(s) to which each category's studies were assigned; studies could map to more than one theme. The two citation-searched foundational studies (Willett 2019; Sharma 2021) were assigned to Theme 2.

Figure S2. Inductive coding progression from 96 initial codes through 8 intermediate categories to the five final themes. Edges indicate the theme or themes to which each category's studies were assigned; studies could be assigned to more than one theme.

Screening reliability is reported as a post hoc check: two members of the research team independently re-screened a random sample of 50 records against the inclusion criteria (Cohen's κ = 0.56; see Section 3.1 and Supplementary S1). The inductive theme-coding of the retained corpus was reached by team consensus rather than parallel independent double-coding, consistent with interpretive synthesis, and is reported without a separate coefficient.

S4. The Opportunity Map: Meta-Jobs and the 90 Strategic Opportunity Areas

Each of the five themes was decomposed into three meta-jobs, yielding fifteen in total (Table S4). Each meta-job was then expressed in each of the six value chain segments, in the order input manufacturing, input distribution, agricultural production, product processing and handling, food manufacturing, and support services, producing ninety Strategic Opportunity Areas. Table S5 reports the bounded opportunity definition for all ninety SOAs, organized by meta-job.

Theme	Meta-jobs
T1 Food quality, nutrition, and health outcomes	MJ1 deliver food that supports optimal health outcomes; MJ2 translate nutritional science into scalable innovation; MJ3 improve transparency and traceability of nutritional value from farm to fork
T2 Sustainable agriculture and food systems	MJ4 align agrifood transformation with health and sustainability outcomes; MJ5 advance resilient and regenerative practices that preserve ecosystem and human health; MJ6 embed sustainability metrics into decision-making across actors
T3 Community and systemic factors in food access and health	MJ7 transform supply chains to equitably serve diverse communities; MJ8 build multi-stakeholder coalitions to address barriers to nutritious food access; MJ9 design policy-aligned agrifood innovations for underserved populations
T4 Food as medicine and nutritional interventions	MJ10 integrate agrifood innovation into healthcare and preventive nutrition; MJ11 create scalable supply systems for medically supportive foods; MJ12 mobilize cost-effective, evidence-based nutrition solutions
T5 Consumer behavior and perceptions	MJ13 support informed consumer decisions through transparent practices; MJ14 respond to evolving consumer values around health and quality; MJ15 leverage consumer insights to guide health-oriented innovation

Table S4. The fifteen meta-jobs by theme.

SOA	Segment	Bounded opportunity definition
01	Input manufacturing	Embed consumer health preferences into input product development through genetics, formulations, and trait selection that drive superior health outcomes.
02	Input distribution	Integrate nutritional objectives into distribution via education, strategic product bundling, and digital usage tracking.
03	Agricultural production	Adapt production systems to health, quality, and sustainability expectations through data-driven cultivation, validated crop varieties, and post-harvest innovations.

SOA	Segment	Bounded opportunity definition
04	Processing and handling	Deliver nutrient preservation, traceability, and health-focused product differentiation at scale via software platforms and digital tools.
05	Food manufacturing	Advance minimally processed, nutritionally robust products, avoid harmful reformulations, and lead in transparent nutritional labeling.
06	Support services	Catalyze and operationalize nutritional and food-safety innovation across the chain through finance, consulting, and impact modeling.
07	Input manufacturing	Translate cutting-edge nutritional science into upstream product design using digital tools, scientific collaborations, and bio-optimized input strategies.
08	Input distribution	Drive adoption of health-aligned inputs as trusted partners, using tools to manage portfolios, upskill teams, and close feedback loops.
09	Agricultural production	Equip producers with systems to shift from yield-only models to nutrition-aware production that operationalizes nutritional science at the field level.
10	Processing and handling	Implement nutrition-sensitive standards and methods that preserve and deliver on health-oriented food innovations.
11	Food manufacturing	Systematically embed nutritional science into product development, balancing evidence-based formulation with commercial realities.
12	Support services	Turn nutritional science into a launchpad for innovation, commercialization, and strategic guidance across the agrifood system.
13	Input manufacturing	Provide end-to-end transparency about how inputs affect food's nutritional integrity using labeling, digital systems, and traceable identifiers.
14	Input distribution	Serve as transparency enablers that digitally record, communicate, and verify how products influence nutritional outcomes across the chain.
15	Agricultural production	Trace, validate, and communicate how production practices influence crop nutrition, integrating with broader value-chain transparency systems.
16	Processing and handling	Document, protect, and share the nutritional value of food during the critical transition from farm to manufacturing.
17	Food manufacturing	Create digital systems that trace and validate nutrient sources, transformations, and claims, building trust from input to label.
18	Support services	Anchor nutritional transparency through traceability platforms, audit and certification systems, and integrated distribution monitoring.
19	Input manufacturing	Align innovation strategies with both environmental sustainability and human health, building safer, cleaner, more equitable food systems.
20	Input distribution	Increase access to safer technologies, enable responsible use, and support downstream health transparency.
21	Agricultural production	Align environmental practices with health outcomes, reducing harm, improving food safety, and supporting farm and community well-being.
22	Processing and handling	Embed health and sustainability into operational and sourcing strategies, supporting cleaner supply chains and stronger food-safety systems.
23	Food manufacturing	Integrate health and sustainability goals into product design, supply-chain strategy, and consumer transparency, strengthening brand equity and reducing risk.
24	Support services	Quantify, report, and manage the health dimensions of agrifood sustainability, bridging public health, environmental science, and enterprise risk.
25	Input manufacturing	Integrate regenerative, ecosystem-stabilizing, and health-preserving principles into product design for responsible agriculture.
26	Input distribution	Catalyze regenerative transitions supporting environmental restoration and human health through bundled offerings, localized engagement, and education.
27	Agricultural production	Implement regenerative production systems that improve ecosystem function while supporting food safety, nutrition, and human health outcomes.
28	Processing and handling	Reinforce the health and environmental benefits of regenerative systems through aligned sourcing, infrastructure innovation, and shorter supply chains.
29	Food manufacturing	Retain and communicate the health and sustainability value of regenerative systems, from formulation to brand positioning.
30	Support services	Quantify and finance the health co-benefits of regenerative agriculture, unlocking new channels for investment, credibility, and impact.
31	Input manufacturing	Integrate health-aligned environmental metrics into sustainability tools and contribute to industry-wide transparency systems.
32	Input distribution	Become advisors and risk mitigators by integrating health-aligned sustainability metrics into digital tools and customer decision support.
33	Agricultural production	Integrate human-health dimensions into sustainability practices and reporting, enhancing compliance, competitiveness, and community relations.
34	Processing and handling	Measure, manage, and report environmental sustainability and associated health impacts comprehensively and transparently.

SOA	Segment	Bounded opportunity definition
35	Food manufacturing	Embed integrated environmental and health-related sustainability metrics within procurement, product development, and brand communication.
36	Support services	Integrate environmental and public-health metrics into reporting, advisory, and evaluation processes.
37	Input manufacturing	Design, produce, and distribute affordable, smallholder-adapted inputs while participating in equitable and inclusive procurement programs.
38	Input distribution	Expand equitable access to inputs, driving health and sustainability impacts in underserved communities.
39	Agricultural production	Provide technical support, crop-selection strategies, and market access tailored to equitably serve food-insecure and culturally diverse communities.
40	Processing and handling	Enhance food access, reduce spoilage, and support remote-community nutrition through innovative infrastructure, technologies, and processes.
41	Food manufacturing	Reformulate, repackage, and reprioritize offerings to align with community nutrition needs while maintaining market viability and scalability.
42	Support services	Mobilize financial, technical, and logistical capacity to redesign supply chains delivering nutrient-rich foods to underserved communities.
43	Input manufacturing	Support food-access coalitions through collaborative product design, joint demonstrations, and coalition-aligned partnerships.
44	Input distribution	Leverage networks, logistics, and technical-service teams in support of regional food-access coalitions as supply-chain coordinators and advisors.
45	Agricultural production	Integrate with place-based coalitions, using land, knowledge, and infrastructure to co-develop localized food-access systems.
46	Processing and handling	Become enablers of nutrition-aligned infrastructure through participation in multi-stakeholder coalitions linking farm to community.
47	Food manufacturing	Participate in cross-sector coalitions supporting positive nutrition outcomes through collaborative product development, engagement, and advocacy.
48	Support services	Coordinate collaborative ecosystems that bridge food-access gaps through technology, advisory services, and systems design for collective impact.
49	Input manufacturing	Shape agronomic innovations supporting nutrition-sensitive food policy by designing tools that make compliance practical, cost-effective, and scalable.
50	Input distribution	Connect upstream innovations to downstream policy objectives by aligning input recommendations with nutrition goals and embedding traceability.
51	Agricultural production	Respond to policy-aligned procurement and food-security initiatives through digitally enabled planning, documentation, and distribution.
52	Processing and handling	Operationalize nutrition-forward food policies by translating guidance into product attributes and standards using compositional analytics.
53	Food manufacturing	Design products and systems compatible with the operational and regulatory expectations of nutrition-focused public programs.
54	Support services	Align agrifood innovation with public nutrition policy through compliance tracking, grant facilitation, and measurable impact reporting.
55	Input manufacturing	Align upstream innovation with healthcare-integrated food systems by embedding health priorities into R&D and certifying clinical-sourcing compatibility.
56	Input distribution	Contribute to food-is-medicine-aligned systems through precision input delivery, digital traceability, and partner coordination.
57	Agricultural production	Meet the quality, traceability, and consistency requirements of healthcare-integrated food systems via precision nutrient management and interoperable data.
58	Processing and handling	Integrate healthcare-ready processing to supply clinically relevant ingredients and intermediates for medically supportive food systems.
59	Food manufacturing	Integrate therapeutic nutrition principles into product design, development, and delivery in partnership with healthcare systems.
60	Support services	Build digital and logistical infrastructure linking agrifood supply with healthcare, including claims management, EHR interoperability, and secure distribution.
61	Input manufacturing	Support food-is-medicine-aligned production through performance validation, compliance, and cost-effective scalability.
62	Input distribution	Enable nutrition-aligned input guidance, coordinated production planning, and early-stage compliance data for medically supportive supply chains.
63	Agricultural production	Align cropping systems, timelines, and quality protocols with food-is-medicine market requirements through planning, compliance tracking, and market access.
64	Processing and handling	Align processing and handling with medically supportive food needs via nutrient tracking, batch control, bioactive validation, and compliance.
65	Food manufacturing	Enable scalable production of clinically relevant, nutritionally validated foods bridging food science, health-system procurement, and packaging technology.

SOA	Segment	Bounded opportunity definition
66	Support services	Provide infrastructure for scalable, consistent distribution of medically supportive foods, integrating health and food data and streamlining delivery.
67	Input manufacturing	Position products within food-is-medicine systems through validated health impact, trial participation, and policy-aligned reimbursement pathways.
68	Input distribution	Connect input choices to measurable nutrition outcomes and support data-sharing with healthcare stakeholders.
69	Agricultural production	Support producers in aligning growing decisions with health outcomes through nutrient-based guidance, practice-outcome tracking, and verified-data incentives.
70	Processing and handling	Integrate health-focused metrics, data systems, and capital-planning tools for evaluating nutritional tradeoffs and health-infrastructure returns.
71	Food manufacturing	Develop validated, cost-effective products supported by healthcare payers by bridging food innovation with clinical trials and payer collaboration.
72	Support services	Enable agrifood and healthcare stakeholders to jointly adopt and validate nutrition-based treatment strategies through outcome and cost-savings modeling.
73	Input manufacturing	Lead data-rich product ecosystems tracing attributes from origin to outcome via transparency features, verification, and partner communication tools.
74	Input distribution	Integrate product-level education, sales training, and digital traceability to become trusted partners in verified health-focused production.
75	Agricultural production	Help producers structure and communicate verified production data, aligning on-farm practices with health-motivated value chains and retail messaging.
76	Processing and handling	Operationalize transparency and maintain product integrity through digital accountability for nutrition, allergen, and origin claims.
77	Food manufacturing	Align product innovation with health evidence, dietary standards, and consumer expectations across formulation and labeling.
78	Support services	Provide digital infrastructure, benchmarking, and transparency tools that help firms translate health and transparency demands into strategy.
79	Input manufacturing	Reposition product strategies in response to evolving consumer health, quality, and sustainability values through agile formulation and portfolio planning.
80	Input distribution	Anticipate and respond to consumer-driven production changes by curating inputs, monitoring demand trends, and offering traceable bundles.
81	Agricultural production	Align production with evolving consumer values through tools enabling data capture, identity preservation, and participation in health-forward networks.
82	Processing and handling	Meet rising expectations for certification alignment, quality signaling, and traceable crop identities, becoming drivers of consumer trust and value.
83	Food manufacturing	Drive consumer-responsive innovation using behavioral analytics, lifestyle segmentation, and agile reformulation.
84	Support services	Translate complex consumer health signals into strategic and operational guidance for agrifood clients across segments.
85	Input manufacturing	Align product innovation with consumer-driven health trends, integrating end-market insights into trait selection, formulation, and go-to-market.
86	Input distribution	Align offerings with wellness trends as strategic advisors, integrating consumer insights into sales, planning, and inventory.
87	Agricultural production	Translate consumer health trends into operational decision support through market intelligence, segmentation insights, and supply-chain connectivity.
88	Processing and handling	Differentiate by responsiveness to health-motivated consumer trends through operational intelligence, traceability, and product segmentation.
89	Food manufacturing	Operationalize consumer insights as a core competency, connecting behavior data to product development, marketing, and testing.
90	Support services	Act as data integrators and innovation catalysts, turning scattered consumer health insights into targeted strategies for the whole chain.

Table S5. Bounded opportunity definition for the ninety Strategic Opportunity Areas, organized by meta-job (SOA 01 to 90).

S5. Discovery Forum Participant Profile

The forum was attended by 37 senior agrifood professionals drawn from across the value chain. The profile below is aggregated and de-identified; categories are author classifications based on organization type and job title, since the roster carries no native segment or seniority field, and counts at category boundaries are approximate. No organization-size information was available. Seven of the 37 served as focal panelists in the structured discovery sessions: four food manufacturing executives and three agricultural producers.

Sector represented	n
Agricultural inputs and crop science	4

Sector represented	n
Agricultural equipment and machinery	3
Agricultural production (farms)	4
Cooperatives, grain handling, and milling	5
Food and ingredient manufacturing	4
Agricultural finance	3
Early-stage ventures and advisory	8
Industry associations, government, and other	6

Table S6. Participants by sector represented (n = 37). Classification is approximate and based on organization type.

Role and seniority	n
C-suite, president, founder, or owner	14
Vice president, director, or principal	17
Manager, lead, analyst, advisor, or other	6

Table S7. Participants by role and seniority (n = 37). Classification is approximate and based on job title.

S6. Discovery Forum Session Protocol

The forum ran over two days. Each day opened with a keynote and a plenary practitioner panel, one panel of food manufacturers and one of agricultural producers, followed by the focal discovery sessions described below, which are the primary instrument for the convergence analysis.

Each of the six focal discovery sessions ran ninety minutes and followed a documented facilitator protocol structured around an industry panelist and a physical Value Proposition Canvas; two of the six sessions, one per day, paired two panelists from the same segment. Participants were divided into three rotating subgroups. In each twenty-minute round, one subgroup conducted a structured Jobs-to-Be-Done interview with the panelist while the other two recorded jobs, pains, and gains on color-coded notes placed on the canvas. Within each round, roughly eight to ten minutes addressed the focal job, five to six minutes the related pains, and four to five minutes the related gains. A facilitator managed timing and labeled each contribution aloud so that it was captured in the recording.

After the three rounds, a twenty-minute consolidation closed the session: a walk-through of the clustered notes, panelist validation and clarification, narrative construction connecting the insights, integration of the clusters into the recorded transcript, and identification of the top three jobs, pains, and gains. All sessions were audio-recorded and transcribed. The interview discipline followed the customer-inquiry principles of the Mom Test (Fitzpatrick, 2013): questions were anchored in specific past behavior, probed real workarounds and costs, and avoided hypothetical or leading questions about whether the practitioner would use a proposed solution. Canvas data and transcripts from the six focal sessions were then coded inductively using the same multi-round procedure as the systematic review (Thomas, 2006).

S7. Value Proposition Canvas Structure

The discovery sessions populated the customer-profile side of the Value Proposition Canvas (Osterwalder et al., 2014), comprising three categories. Customer jobs were recorded as functional, social, and emotional jobs. Customer pains covered obstacles, risks, and undesired outcomes. Customer gains covered required, desired, and unexpected gains. The value-map side of the canvas was not populated, consistent with a discovery objective focused on eliciting practitioner needs rather than evaluating solutions. Completed canvases contain participant and company identifiers and are therefore not reproduced; the consolidated, de-identified jobs, pains, and gains for each primary segment appear in Table 3 of the main text.

S8. AI Capability Assessment: Procedure and Evidence Base

Section 5 of the main text assesses how far current artificial intelligence can address the twelve prioritized opportunities. The procedure was as follows. For each prioritized SOA, the functional requirement implied by its bounded opportunity definition was matched to one or more of five AI capability families: C1 sensing, measurement, and prediction; C2 traceability and verification; C3 evidence synthesis and regulatory intelligence; C4 generative and predictive design; and C5 personalized nutrition and decision support. Peer-reviewed evidence for each match was identified through targeted searches of the food-science, agricultural, biomedical, and computing literatures, giving preference to recent review and systematic-review articles.

Every reference used in the assessment was verified against the Crossref and Europe PMC metadata records for the canonical publisher version; preprints and non-scholarly sources were excluded. Each opportunity was then graded strong, where multiple peer-reviewed reviews document mature and validated methods; moderate or caveated, where the literature is active but reports material reliability limits; or not primarily AI-addressable, where the binding constraint is relational or market-structural.

The AI literature was identified through structured searches of Scopus, PubMed, Web of Science, and Google Scholar conducted in June 2026, combining terms for artificial intelligence and its sub-fields (machine learning, deep learning, large language models, computer vision, and blockchain) with terms for each capability family and the food or agricultural domain. Priority was given to peer-reviewed review and systematic-review articles published from 2020 onward in established journals; preprints, conference abstracts, and trade material were excluded. Candidate sources were screened for direct relevance to a prioritized opportunity, and every retained source was verified against its Crossref and Europe PMC metadata record before inclusion. Twenty peer-reviewed sources were retained as the evidence base for the assessment and are cited in Sections 2.4 and 5 of the main text.

Capability family	Prioritized SOAs addressed	Evidence	Key reliability caveat
C1 Sensing, measurement, and prediction	2, 7, 9, 10, 12	Strong	Adoption and integration, not technical feasibility, is the constraint
C2 Traceability and verification	1, 4, 12	Strong	Verifies only what is measured and recorded; depends on data sharing
C3 Evidence synthesis and regulatory intelligence	1, 3, 5, 6	Moderate / caveated	Hallucination and unreliable risk-of-bias assessment; requires human oversight
C4 Generative and predictive design	8, 10	Strong	Model outputs require experimental validation before market
C5 Personalized nutrition and decision support	3, 11	Strong	Clinical deployment carries added regulatory and validation requirements

Table S8. The five AI capability families, the prioritized SOAs each addresses, the strength of the peer-reviewed evidence, and the principal reliability caveat. The long-term-contract and market-creation constraints identified in the forum are not addressable by any family (Section 5.6).

The peer-reviewed sources underpinning each capability family are cited in Section 2.4 and Section 5 of the main text and appear in the main reference list. The use of a generative artificial intelligence tool in preparing this manuscript is disclosed in the Acknowledgments of the main text.

S9. Methodological Quality Appraisal of the Included Studies

We appraised the methodological quality of the 34 included studies using tools matched to each study's design. Empirical studies (quantitative, qualitative, and mixed methods) were appraised with the Mixed Methods Appraisal Tool (MMAT, 2018 version), which applies two screening questions and five design-specific criteria. Studies that were themselves systematic or literature reviews were assessed for whether they reported a search strategy, explicit selection criteria, and an appraisal of their own included studies. Items that were editorials, special-issue introductions, consensus or advisory statements, commission reports, or professional commentary were classified as non-empirical and retained as contextual evidence rather than formally scored, consistent with the inclusion criteria, which admitted established institutional reports alongside empirical studies and reviews.

Of the 34 studies, 12 are empirical, 5 are reviews (one a registered systematic review), and 17 are non-empirical. Among the twelve empirical studies, the number of MMAT criteria met ranges from 1 to 5 of 5, with a median of 3, so quality is generally moderate: the strongest report validated measurement and appropriate analysis, while the recurring limitations are sample representativeness and selection bias, unaddressed nonresponse or attrition, and incomplete methods reporting. The heterogeneous and largely non-empirical composition of the corpus reflects an applied and fast-moving field, and it bounds the strength of the evidence base, as noted in the main-text limitations (Section 6.3). Table S9 reports the appraisal for each study.

#	First author (Year)	Design class	Appraisal tool	Quality note
1	Johnson et al. (2023)	Qualitative	MMAT 2018 (qualitative)	5/5; small single-clinic sample limits transferability
2	McConnell et al. (2016)	Non-empirical	Not scored	Symposium introduction; contextual
3	Ylitalo et al. (2024)	Quantitative descriptive	MMAT 2018 (quant. descriptive)	3/5; satisfaction data from a self-selected refill minority; physician response rate unreported
4	Li et al. (2024)	Narrative review	Review appraisal	Perspective/narrative review; no systematic protocol
5	Clark et al. (2022)	Non-empirical	Not scored	Program description; contextual
6	Guardiola-Márquez et al. (2022)	Narrative review	Review appraisal	Biofortification review; no systematic protocol
7	Roe (2025)	Non-empirical	Not scored	Outreach article; contextual

#	First author (Year)	Design class	Appraisal tool	Quality note
8	Bernstein et al. (2024)	Non-empirical	Not scored	Perspective; contextual
9	Ridberg et al. (2024)	Non-empirical	Not scored	Meeting report; contextual
10	Volpp et al. (2023)	Non-empirical	Not scored	AHA presidential advisory (consensus); high-authority context
11	Sollid et al. (2022)	Quantitative descriptive (survey)	MMAT 2018 (quant. descriptive)	1/5; sampling frame, instrument, and response rate unreported
12	Mihafu et al. (2020)	Narrative review	Review appraisal	Narrative review; no systematic protocol
13	Rahman et al. (2024)	Non-empirical	Not scored	Perspective; contextual
14	Muchenje et al. (2018)	Non-empirical	Not scored	Special-issue introduction; contextual
15	Maestre et al. (2018)	Non-empirical	Not scored	Editorial introduction; contextual
16	Kline et al. (2024)	Qualitative	MMAT 2018 (qualitative)	5/5; purposive and snowball sample; analysis procedure reported briefly
17	Vogl et al. (2016)	Qualitative	MMAT 2018 (qualitative)	4/5; category-derivation procedure undescribed
18	Venn (2020)	Non-empirical	Not scored	Editorial; contextual
19	Mattoo et al. (2023)	Quantitative, non-randomized (field)	MMAT 2018 (quant. non-randomized)	3/5; single site; unequal yearly sample sets; year confounding
20	Erisman (2021)	Non-empirical	Not scored	Perspective/opinion; contextual
21	Rahmann (2016)	Non-empirical	Not scored	Position paper; contextual
22	Ohno et al. (2018)	Non-empirical	Not scored	Special-section introduction; contextual
23	Higgins et al. (2022)	Qualitative (case study)	MMAT 2018 (qualitative)	2/5; comparative case study; no analysis procedure; little primary data shown
24	Shroff et al. (2020)	Non-empirical	Not scored	Commentary essay; contextual
25	Hristakeva et al. (2025)	Quantitative, non-randomized (DiD)	MMAT 2018 (quant. non-randomized)	2/5; strong matched difference-in-differences design; attrition and adherence unreported; now published (reference 7)
26	Olson (2017)	Quantitative descriptive (content analysis)	MMAT 2018 (quant. descriptive)	3/5; systematic coding with inter-judge reliability; self-selected commenters
27	Choe et al. (2018)	Narrative review	Review appraisal	Microgreens review; no systematic protocol
28	Wadhwa (2020)	Non-empirical	Not scored	Column commentary; contextual
29	Fatemi et al. (2023)	Quantitative descriptive (text analysis)	MMAT 2018 (quant. descriptive)	3/5; large corpus; Twitter users undifferentiated; platform-biased sample
30	Shi et al. (2023)	Quantitative, non-randomized (crossover)	MMAT 2018 (quant. non-randomized)	3/5; very small sample (n = 15); no order randomization reported
31	Tian et al. (2024)	Quantitative descriptive (analytical)	MMAT 2018 (quant. descriptive)	3/5; strong analytical measurement; one harvest year; data completeness and correlation test not stated
32	Fredericks et al. (2024)	Non-empirical	Not scored	Framework perspective; contextual
33	Willett et al. (2019)	Non-empirical	Not scored	EAT-Lancet Commission report (consensus); high-authority context
34	Sharma et al. (2021)	Systematic review	AMSTAR-style review appraisal	Registered PRISMA systematic review; robust (EPHPP/CASP appraisal of its studies)

Table S9. Methodological quality appraisal of the 34 included studies. Empirical studies were appraised with MMAT 2018; reviews with a review-appropriate check; non-empirical items were classified and retained as contextual evidence, not formally scored.

S10. Evidence Base for the Prioritized Opportunities

Table S10 records, for each of the twelve prioritized opportunities, the AI capability family that applies, the strength of the peer-reviewed evidence, the representative supporting literature, the principal reliability or validation limit, and the rationale for the grade. All entries derive from the AI-capability assessment described in Section 5 and above; the grades follow the criteria in Section 3.5.

#	Prioritized opportunity	AI capability family	Grade	Representative literature	Principal reliability / validation limit	Grade rationale
1	Health-claim substantiation	Traceability + evidence synthesis (C2, C3)	Moderate	[14,43]	Evidence-synthesis reliability limits; requires human oversight	Two families; the evidence-synthesis component is caveated
2	On-farm measurement and verification	Sensing and prediction (C1)	Strong	[35,36]	The constraint is adoption and integration; feasibility is established	Multiple reviews document validated methods
3	Evidence-based product platforms	Evidence synthesis + personalization (C3, C5)	Moderate	[11,42]	Evidence-synthesis reliability limits	Mixed; personalization strong, evidence synthesis caveated
4	Farm-to-fork transparency	Traceability and verification (C2)	Strong	[13,40]	Verifies only what is measured; depends on data sharing	Deployment-ready reviews, commercial pilots
5	Cross-jurisdictional regulatory intelligence	Regulatory NLP/LLMs (C3)	Moderate	[41,59]	Hallucination; unsupervised use unsafe	Active literature but documented reliability limits
6	Regulatory intelligence platform	Regulatory NLP/LLMs (C3)	Moderate	[42,59]	Hallucination; requires human oversight	Active literature but documented reliability limits

#	Prioritized opportunity	AI capability family	Grade	Representative literature	Principal reliability / validation limit	Grade rationale
7	Production-system design for nutritional density and premium capture	Sensing and prediction (C1)	Strong	[37,60]	Grade applies to measurement and design; the premium mechanism is not AI-addressable (Section 5.6)	Validated sensing; premium capture is a market function
8	Reformulation platforms	Generative and predictive design (C4)	Strong	[12,45]	Model outputs require experimental validation before market	Mature predictive design; laboratory validation is a required step and does not count as a reliability failure
9	Regenerative transition with health co-benefits	Sensing and dMRV (C1)	Strong	[37,60]	Validated MRV methods; vary with soil type and depth	Remote-sensing/ML MRV documented
10	Seed/input traits for nutritional density	Sensing + generative design (C1, C4)	Strong	[38,39]	Model transfer limited; field validation needed	Genomic selection and model-assisted breeding validated
11	Healthcare-system partnerships	Personalized nutrition and decision support (C5)	Strong	[10,46]	Clinical deployment adds regulatory and validation requirements	Strong and growing outcome-prediction evidence
12	Third-party verification and analytics	Sensing + traceability (C1, C2)	Strong	[13,36]	Depends on upstream sensing and data sharing	Validated sensing plus traceability

Table S10. Per-opportunity evidence base for the twelve prioritized opportunities. Capability families: C1 sensing, measurement, and prediction; C2 traceability and verification; C3 evidence synthesis and regulatory intelligence; C4 generative and predictive design; C5 personalized nutrition and decision support. A strong grade denotes method maturity under study conditions and does not by itself establish commercial readiness, which requires physical testing.

Worked examples of statement-to-SOA assignment.

The convergence analysis assigned each job, pain, and gain from the focal sessions to one or more SOAs by matching its functional content to the recorded opportunity definition. Three examples illustrate the procedure. A producer's description of the absence of integrated on-farm record-keeping ("I would love nothing more than an SAP system for my operation") was assigned to SOA 2 (on-farm measurement and verification of nutritional and health attributes), because its functional content concerns documenting farm-level attributes. A manufacturer's account of more than a thousand products on a multi-tier clean-label transition pathway was assigned to SOA 8 (reformulation platforms balancing nutrition and perceived quality), because its functional content concerns combinatorial reformulation under regulatory and consumer pressure. A manufacturer's report of five or six full-time roles monitoring state-level and customer-specific requirements manually was assigned to SOA 5 (cross-jurisdictional regulatory intelligence for health claims), because its functional content concerns tracking obligations across jurisdictions.
